# GHB toxicokinetics and renal monocarboxylate transporter expression are influenced by the estrus cycle in rats

**DOI:** 10.1186/s40360-023-00700-y

**Published:** 2023-11-02

**Authors:** Hao Wei, Jieyun Cao, Tyler Fallert, Su Yeo, Melanie A. Felmlee

**Affiliations:** 1 Department of Pharmaceutics and Medicinal Chemistry, Thomas J Long School of Pharmacy and Health Sciences, University of the Pacific, Stockton, CA 95211 USA; 2 Present Address: AstraZeneca, Gaithersburg, Maryland USA; 3 Present Address: Clovis Community Medical Center, Clovis, CA USA; 4 Present Address: Kaiser Permanente, Santa Clara, CA USA

**Keywords:** Gamma-hydroxybutyric acid, Monocarboxylate transporters

## Abstract

**Background:**

The illicit use and abuse of gamma-hydroxybutyric acid (GHB) occurs due to its sedative/hypnotic and euphoric effects. Currently, there are no clinically available therapies to treat GHB overdose, and care focuses on symptom treatment until the drug is eliminated from the body. Proton- and sodium-dependent monocarboxylate transporters (MCTs (SLC16A) and SMCTs (SLC5A)) transport and mediate the renal clearance and distribution of GHB. Previously, it has been shown that MCT expression is regulated by sex hormones in the liver, skeletal muscle and Sertoli cells. The focus of the current study is to evaluate GHB toxicokinetics and renal monocarboxylate transporter expression over the estrus cycle in females, and in the absence of male and female sex hormones.

**Methods:**

GHB toxicokinetics and renal transporter expression of MCT1, SMCT1 and CD147 were evaluated in females over the estrus cycle, and in ovariectomized (OVX) female, male and castrated (CST) male rats. GHB was administered iv bolus (600 and 1000 mg/kg) and plasma and urine samples were collected for six hours post-dose. GHB concentrations were quantified using a validated LC/MS/MS assay. Transporter mRNA and protein expression was quantified by qPCR and Western Blot.

**Results:**

GHB renal clearance and AUC varied between sexes and over the estrus cycle in females with higher renal clearance and a lower AUC in proestrus females as compared to males (intact and CST), and OVX females. We demonstrated that renal MCT1 membrane expression varies over the estrus cycle, with the lowest expression observed in proestrus females, which is consistent with the observed changes in GHB renal clearance.

**Conclusions:**

Our results suggest that females may be less susceptible to GHB-induced toxicity due to decreased exposure resulting from increased renal clearance, as a result of decreased renal MCT1 expression.

**Supplementary Information:**

The online version contains supplementary material available at 10.1186/s40360-023-00700-y.

## Background

γ-hydroxybutyrate (GHB), also known as γ-hydroxybutyric acid, 4-hydroxybutyric acid, and 4-hydroxybutanoic acid, is an endogenous four carbon chain fatty acid [1]. GHB is produced from γ-aminobutyric acid (GABA), which acts as a neurotransmitter [2]. GHB is a neuromodulator in the central nervous system, and interacts with the GHB and GABA_B_ receptors [3]. GHB is used clinically as Xyrem (sodium oxybate; Jazz Pharmaceuticals) to treat narcolepsy with cataplexy in America [4], and to treat alcohol withdrawal, heroin dependence, sleep disorders and narcolepsy in Europe [2, 5]. GHB is most famous for its illicit use and abuse in night clubs, raves and drug-facilitated sexual assault, because of its sedative/hypnotic and euphoric effects [1]. GHB is colorless, odorless and tasteless, and has high solubility; therefore, it is easy to unknowingly administer a high dose. The dose-response curve for GHB is very steep; minor increases in dose can lead to significant adverse events. Currently, there are no clinically available therapies to treat GHB overdose and care focuses on symptom treatment until the drug is cleared from the body.

Monocarboxylate transporters have been found to transport and mediate GHB disposition [6–8]. There are 14 members in the proton-dependent monocarboxylate transporter family (MCT; SLC16) with MCT1, MCT2 and MCT4 demonstrated to transport GHB [8, 9]. Additionally, GHB is transported by sodium-dependent monocarboxylate transporters (SMCTs; SLC5A8 and SLC5A12) [10]. MCTs and SMCTs are expressed in the renal proximal tubules [10–12]. MCTs are expressed on the basolateral surface of epithelial cells in the proximal tubules of kidney while the SMCTs are expressed at the apical membrane [13]. CD147 is an ancillary protein responsible for MCT membrane trafficking and localization, due to the presence of a basolateral sorting sequence [14, 15]. SMCTs are localized to the apical membrane of renal proximal tubules [13], with SMCT1 expressed in the S3 segment of proximal tubule, while SMCT2 is expressed on the entire length of the proximal tubule in kidney [16]. Based on their different membrane localization, MCTs and SMCTs co-operate in the active reabsorption of GHB in the kidney. Variations in MCT/SMCT expression in the kidney lead to changes in transport capacity, which have the potential to impact GHB renal clearance through alterations in active renal reabsorption [17]. Increased expression of MCTs/SMCTs would increase renal reabsorption of GHB, thereby increasing systemic plasma concentration and the potential for toxicity.

Literature data demonstrates that some MCT substrates display sex differences in systemic plasma concentrations in both human and rodents. Cupeiro et al. (2012) demonstrated that males have higher plasma lactate levels following exercise [18]. This sex difference could be the result of differences in transporter capacity in muscle. In a study of valproic acid pharmacokinetics, women had higher peak plasma concentrations compared to men, unless they were on hormonal birth control [19], which may be a result of sex differences in MCT/SMCT expression. Both progesterone and estrogen down-regulated renal SMCT1 protein expression in female mice [20]. SMCT1 protein levels were increased by orchiectomy and testosterone replacement recovered the change in male mice [21]. Testosterone treated rats demonstrated higher lactate transport into giant sarcolemmal vesicles [22]. It had been demonstrated that monocarboxylate transporters and their ancillary protein, CD147, are regulated by sex hormones in a tissue specific manner [23, 24]. We previously demonstrated that monocarboxylate transporters are regulated by sex hormones in rat liver with variation over the estrus cycle in females [25]. However, currently there is no literature data evaluating if GHB toxicokinetics are influenced by sex hormones or differ between sexes.

The objective of the present study was to quantify GHB toxicokinetics and renal monocarboxylate transporter expression over the estrus cycle in females, and in the presence and absence of male and female sex hormones. In the present study, toxicokinetics and renal clearance were evaluated following 600 mg/kg and 1000 mg/kg iv bolus GHB administration in males, females over the estrus cycle, and in the absence of sex hormones (through ovariectomy and castration). Renal expression of MCT1, SMCT1 and the ancillary protein CD147 was evaluated over the estrus cycle in females, in males, and in the absence of female or male sex hormones in Sprague-Dawley rats.

## Methods

### Chemicals and reagents

GHB (sodium salt) was obtained from the NIDA Drug Supply Program. Formic acid was purchased from Fisher Scientific (Fair Lawn, NJ). Deuterated GHB (GHB-d6) was obtained from Sigma-Aldrich (St. Louis, MO). Ketamine, xylazine, and heparin were purchased from Patterson Veterinary (Saint Paul, MN). High-performance liquid chromatography (HPLC)-grade acetonitrile, methanol, and acetic acid were purchased from Fisher Scientific (Fair Lawn, NJ).

### Animals and estrus staging

Male and female Sprague Dawley (SD) rats, ovariectomized (OVX) females and castrated (CST) males were obtained from Charles River (Wilmington, MA) at 8 weeks of age and were housed in a temperature-controlled room with a 12-hour day/night lighting cycle. Room temperature were at 25 °C with steady humidity. All rats were fed standard rat chow and water *ad libitum*. Animals were acclimatized for at least one week before experiments. The estrus stage of female rats was monitored daily by vaginal lavage smear for 8–12 days (at least two estrus cycle) between 10 am and 12 pm. Vaginal lavage procedures and estrus cycle assignments were performed as previously described [25–27]. The Institutional Animal Care and Use Committee at the University of the Pacific approved all experiments.

### Toxicokinetic study

A jugular vein cannula was implanted in all animals for drug administration and blood sampling. Surgery was performed at least two days before the toxicokinetic studies. GHB (600 or 1000 mg/kg) was administered by intravenous bolus injection via the jugular vein cannula to male, different estrus cycle stage females, OVX females and CST males at 10 weeks of age (N = 4–5 per group). Rats were dosed in metabolic cages with *ad libitum* access to water to allow for the simultaneous collection of blood and urine samples for 6 h. Blood samples (200 µl) were taken from the jugular vein cannula at different time points 10, 30, 60, 90, 120, 180, 210, 240 and 270 min for 600 mg/kg and 5, 10, 20, 30, 60, 90, 120, 180, 240, 270, 300 and 330 min for 1000 mg/kg, and transferred into heparinized 0.65 ml microcentrifuge tubes. Plasma samples were separated by centrifugation at 5000 g for 15 min at 4℃ and stored at -20℃. Urine samples were collected at 120, 240, and 360 min post-dose, the total urine volume was determined, and the samples were transferred into 15 ml centrifuge tubes and stored at -20℃.

### LC/MS/MS assay

GHB plasma sample preparation followed a previously published method with minor modifications [4, 28]. Briefly, GHB standard stock solutions were prepared in double-distilled water at the following concentrations: 50, 100, 250, 1250, 2500, 5000 µg/ml. Quality control stock solutions were prepared separately at 100 and 5000 µg/ml for low QC and high QC, respectively. For standards and quality controls, 5 µl of GHB-d6, 5 µl of standard stock solution or QC stock solution was combined with 45 µl of blank plasma. For each unknown plasma sample, 5 µl of GHB-d6 was added to 50 µl plasma. 800 µl of acetonitrile with 0.1% formic acid was added to all samples and standards to precipitate plasma proteins followed by centrifugation at 10,000 g for 20 min. 750 µl of the supernatant was removed, transferred to a clean microcentrifuge tube and evaporated by using CentriVap Concentrator (Labconco, Kansas City, MO) under vacuum status [600 mg/kg dose] or using a MultiVap Nitrogen Evaporator (Organomation, Berlin, MA) under a nitrogen stream [1000 mg/kg dose]. Dried samples were reconstituted in 250 µl of 95:5 ddH_2_O: acetonitrile with 0.1% acetic acid.

Urine sample preparation followed a previously published method with minor modifications [4, 28]. GHB standard stock solutions for urine samples were prepared in double-distilled water at the following concentrations: 20, 100, 200, 400, 1000, 2000 µg/ml. Quality control stock solutions were prepared separately at 20, 200 and 1000 µg/ml (in double-distilled water) for QC-low, QC-mid and QC-high, respectively. For standards and QCs, 25 µl of blank urine, 5 µl of standard or QC stock solution, 5 µl of GHB-d6 (500 µg/ml) and 465 µl ddH_2_O were combined. For unknown urine samples, 25 µl sample, 5 µl GHB-d6 (500 µg/ml) and 470 µl ddH_2_O.

GHB concentrations in plasma and urine samples were quantified using a validated LC/MS/MS assay described previously with minor modifications [4]. The LC/MS/MS system included an Agilent 1100 series HPLC consisting of an online degasser, binary pump, and autosampler (Agilent Technologies, Santa Clara, CA) connected to MDS Sciex API 3000 triple – quadruple tandem mass spectrometer equipped with a turbo ion spray (Applied Biosystems, Foster City, CA) [600 mg/kg dose] or the LC/MS system included an Agilent 1200 series UPLC consisting of online degasser, binary pump, and autosampler (Agilent Technologies, Santa Clara, CA) connected to Agilent 6460 Triple Quad Mass Spectrometer [1000 mg/kg dose]. 20 µl of sample was injected into an Xterra MS C18 column (250 × 2.1 mm i.d., 5-µm particle size; Waters, Milford, MA). Mobile phase A was 0.1% formic acid in double-distilled water with 5% acetonitrile. Mobile phase B was 0.1% formic in acetonitrile with 5% double-distilled water. A gradient elution with a flow rate of 200 µl/min was used to separate compounds: 100–90% A over 5 min, 90–10% A over 2.5 min, and 10–100% A over 4.5 min with a total run time of 12 min. The mass spectrometer was operated in positive ionization mode with multiple reaction monitoring. Tables 1 and 2 show the details of the mass spectrometer parameters for GHB and GHB-d6. The retention time for GHB and GHB-d6 was 3.57 min and the standard curve in plasma ranged from 5 to 500 µg/mL and in urine from 2 µg/mL to 400 µg/mL. Inter- and intra-day accuracy and precision based on quality control samples of the analyte were 100 ± 10%.

**Table 1:** Mass spectrometer parameters of API3000 for MRM analysis of GHB.

Parameters	GHB	GHB-d_6_
Q1/Q3	105/87	111.2/93
Declustering potential (volts)	6	11
Collision energy (volts)	9	11
Collision cell exit potential (volts)	6	16

**Table 2:** Mass spectrometer parameters of Triple Quad for MRM analysis of GHB.

Parameters	GHB	GHB-d_6_
Q1/Q3	105.1/87.1	111.1/93.1
Fragmentor (volts)	55	45
Collision energy (volts)	4	4
Cell acceleration voltage (volts)	4	4

### Tissue collection

Kidney samples were collected from a second group of male and female rats at 10 weeks of age following exsanguination under isoflurane anesthesia between 10 am to 12 pm (N = 3–5 per group). Vaginal lavage was performed immediately before sacrificing to confirm females’ estrus stage, and each estrus stage evaluated represents an independent group of animals. Age-matched OVX female and CST male samples were collected two weeks post-surgery. Tissue samples were immediately snap frozen in liquid nitrogen and stored at -80 °C until analysis.

### qPCR

Total RNA was isolated from frozen tissue (~ 30 mg) using a PureLink RNA Mini Kit (ThermoFisher Scientific) following the manufacturer’s instructions. RNA concentration and quality was determined by Nanodrop (Thermo Fisher Scientific). FlashGel RNA cassettes (Lonza) were used to verify the integrity of all RNA samples prior to qPCR analysis. RNA integrity was demonstrated by the presence of two clear bands representing 28 S and 18 S rRNA with an intensity ratio of 2:1 [29]. 2000 ng of total RNA was added to the cDNA synthesis reaction (5x iScript, BioRad). For each reaction, 1 µL of cDNA, 1 µL of 5 µM forward and reverse primers were used. Primer sequences, annealing temperatures, PCR product sizes and assay validation were previously described [25]. Serial dilutions of plasmids containing the gene-specific amplicon were used as standards to evaluate qPCR efficiency and quantify relative gene expression. Quantitative analysis of MCT1, SMCT1, CD147 and Alien RNA (external control) were performed with iTaq Universal SYBR Green Supermix as previously described [25]. All biological samples were run in triplicate and data was quantified by the ΔΔC_T_ method or in arbitrary units based on the standard curve.

### Western blot

Sample preparation and western blot analysis was previously validated in our laboratory [25, 30]. Kidney tissue (20–30 mg) was homogenized in 600 µL ice-cold radio-immunoprecipitation assay buffer (RIPA) containing Protease Inhibitor Cocktail (UltraCruz, Santa Cruz Biotechnology), the ratio between the mass of the tissue (in ug) and the volume of the buffer (in uL) is 33.3:1 to 50:1, to obtain the whole cell protein extract. The sample was centrifuged at 16,000 × g for 15 min at 4 °C to remove cell debris. The supernatant was retained for analysis of whole cell protein expression and stored at -20 °C until analysis. Membrane bound proteins were extracted from kidney using a ProteoExtract Native Membrane Extraction kit (EMD Millipore) following the manufacturer’s instructions. Na^+^/K^+^ ATPase enrichment in the membrane fraction of the sample indicated successful membrane isolation. Protein concentrations were determined using a Pierce BCA Protein Assay Kit (ThermoFisher) with bovine serum albumin as the standard.

Total whole cell protein (15 µg) or membrane fraction (5 µg) were mixed with 2x and 4x Laemmli sample buffer (BioRad) and heated at 37 °C for 30 min. Samples were separated at 200 V on 10% TGX Fast Cast gel (BioRad) and transferred to nitrocellulose membranes at 100 V for 25 min. GAPDH and Na^+^/K^+^ ATPase were visualized using a Chemidoc Touch Imaging System (BioRad) to normalize expression. Membranes were incubated in SEA Blocking buffer (Thermo Fisher Scientific) or 5% milk in PBST for 1 h (MCT1 and CD147) or 1.5 h (SMCT1) at room temperature. Membranes were incubated with primary antibody on a shaker overnight at 4 °C. Primary antibodies were rabbit anti-MCT1 (AB3540P, EMD Millipore) at a 1:1350 dilution, goat anti-CD147 (sc-9757, Santa Cruz Biotechnology) at a 1:1000 dilution, rabbit anti-SMCT1 (ARP44110_P050, Aviva Systems Biology) at a 1:1000 dilution, rabbit anti- Na^+^/K^+^ ATPase (sc-28,800, Santa Cruz Biotechnology), mouse anti-GAPDH (sc-32,233, Santa Cruz Biotechnology) at a 1:1000 dilution. Secondary antibodies were anti-goat (ab97110, Abcam), anti-mouse (sc-516,102, Santa Cruz Biotechnology) anti-rabbit IgG (ab97051, Abcam) at a 1:10,000 dilution. The membranes were then incubated with Clarity ECL reagent (BioRad) for 2 min (MCT1 and CD147) or Radiance Plus reagent (Azure Biosystems) for 3 min (SMCT1). Protein bands were visualized using a Chemidoc Touch Imaging System (BioRad). Band density was determined using BioRad Image lab software with Na^+^/K^+^ ATPase as a loading control for membrane protein and GAPDH as loading control for total protein. Samples were analyzed individually by western blot.

### Data analysis

All data are expressed as mean ± standard deviation. Non-compartmental analysis of the plasma concentration-time profiles was conducted in Phoenix WinNonLin (Certara) to obtain AUC_inf_ and total clearance (CL). Cumulative urinary excretion of GHB was calculated by summing the amount excreted in urine for all collection intervals. The fraction of drug eliminated in the urine (f_e_) is the quotient of cumulative excretion divided by the product of dosage and body weight (f_e_ = A_e,inf_/(Dose*BW)). Renal clearance (CL_R_) was calculated as the total amount excreted in urine divided by the plasma AUC (CL_R_ = A_e,inf_/AUC). Metabolic clearance (Cl_m_) was calculated as the difference of total clearance and renal clearance [CL_m_ = CL – CL_R_]. Expression of mRNA was normalized to the mean of the house-keeping gene 18 S and Alien RNA, and fold-change was determined using the ∆∆C_T_ method. MCT1, SMCT1 and CD147 whole cell protein expression was normalized to GAPDH. MCT1, SMCT1 and CD147 membrane protein expression was normalized to Na^+^/K^+^ ATPase expression. Statistical differences were determined in GraphPad Prism 7 using an unpaired t-test (male data set) or one-way analysis of variance (ANOVA) with a Tukey’s post hoc test (female data set, combined data sets). Differences were considered statistically significant when the P value was less than 0.05.

## Results

### Estrus staging

**Fig. 1:**
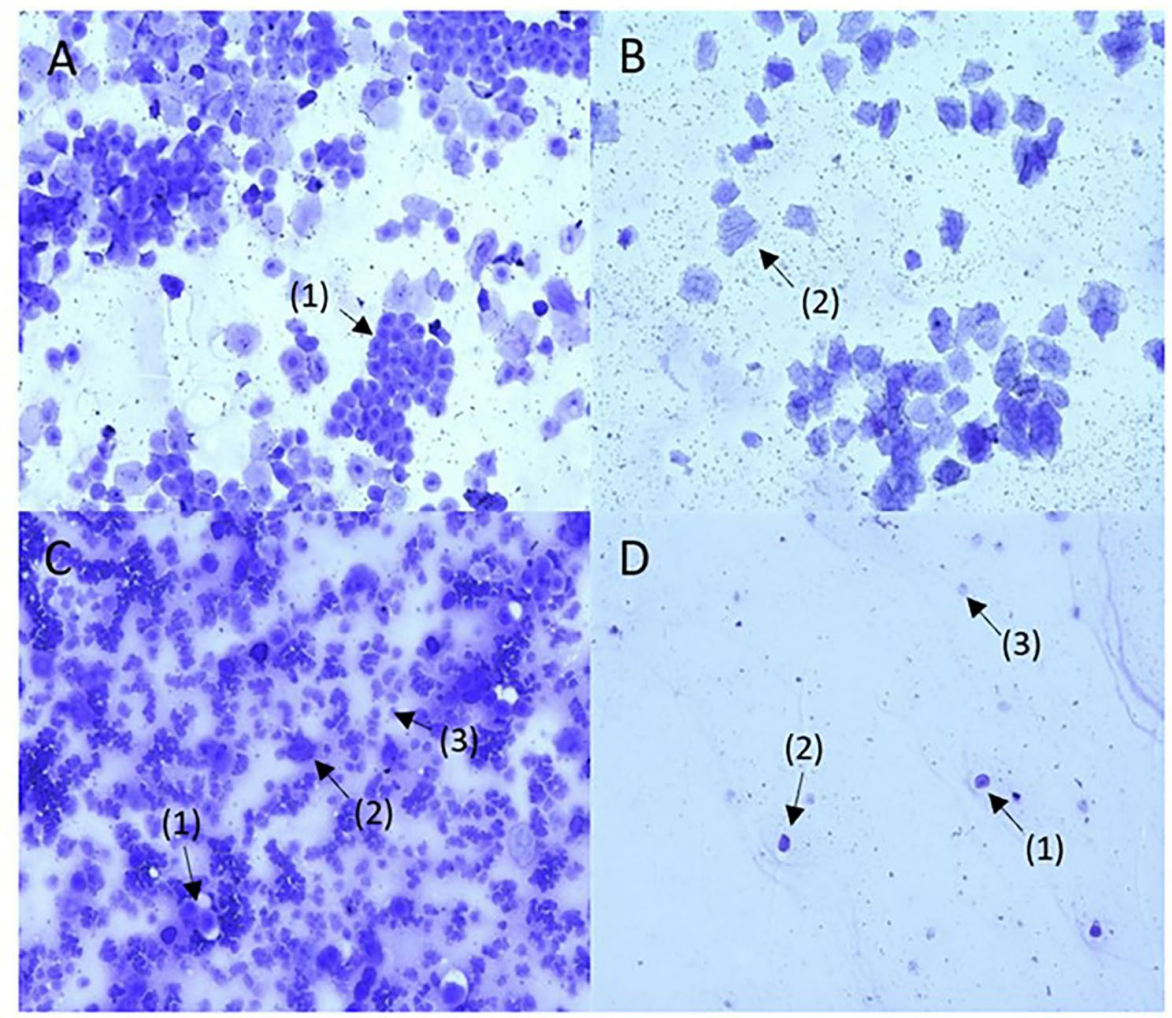
Representative vaginal smears for the four estrous cycle stages in female Sprague-Dawley rats at 20 times magnification. [**A**]. Proestrus characterized by large nucleated epithelial cells [1]. [**B**] Estrus characterized by large anucleated keratinized epithelial cells [2]. [**C**] Metestrus characterized by a combination of epithelial cells and neutrophils [3]. [**D**] Diestrus characterized by low cellularity, and a combination of neutrophils, small and large epithelial cells [1–3]

Female estrus groups were assigned based on vaginal lavage smears. Representative smear images are shown in Fig. 1.

### Toxicokinetic analysis

Non-compartmental analysis of the plasma concentration time profiles was conducted in Phoenix WinNonLin and the results are presented in Tables 3 and 4. Total clearance was influenced by sex and the estrus cycle, with proestrus and estrus females trending higher than other groups at 600 mg/kg. Total clearance was significantly higher in proestrus, metestrus and diestrus females at 1000 mg/kg, as compared to OVX females and male rats. Interestingly, total clearance values increased for the 1000 mg/kg dose in proestrus, metestrus and diestrus females, while all remaining groups had consistent clearance values. The total clearance results are consistent with the calculated AUC values: groups with higher clearance values had lower AUCs. The AUC of OVX and males (intact and CST) were higher than all estrus stages at both doses. There was no difference in clearance and AUC values between intact and CST males.

**Table 3:** Toxicokinetic parameters following iv administration of 600 mg/kg GHB in females over the estrus cycle, ovariectomized females (OVX), males and castrated (CST) males. Data is presented as mean ± SD, N = 4–5. ^*^P < 0.05 when compared to proestrus females. ^#^P < 0.05 when compared to estrus females

Parameter	Units	Proestrus	Estrus	Metestrus	Diestrus	OVX	Male	CST
AUC	mg*min/mL	89 ± 16.8	93 ± 18.3	108 ± 16.3	129 ± 16.8	120 ± 35.5	115 ± 21.6	116 ± 17.1
CL	mL/min/kg	6.8 ± 2.6	6.7 ± 2.5	5.6 ± 2.1	4.7 ± 1.8	5.3 ± 2.1	5.4 ± 2.1	5.3 ± 2.1
CL_R_	mL/min/kg	3.7 ± 0.4	3.3 ± 0.09	2.6 ± 0.3	1.9 ± 0.7^*#^	1.9 ± 1.1^*#^	2.0 ± 1.0^*^	2.1 ± 0.8^*^
f_e_		0.54 ± 0.03	0.49 ± 0.09	0.47 ± 0.05	0.40 ± 0.07	0.36 ± 0.1^*^	0.38 ± 0.06	0.39 ± 0.03
CL_m_	mL/min/kg	3.1 ± 0.4	3.4 ± 0.7	3.0 ± 0.6	2.9 ± 0.6	3.4 ± 1.1	3.4 ± 1.1	3.2 ± 0.5

AUC - Area under the curve of plasma concentration versus time profile

CL - Total clearance

CL_R_ - Renal clearance

f_e_ – The fraction of drug eliminated from urine

CL_m_ – Metabolic clearance

**Table 4:** Toxicokinetic parameters following iv administration of 1000 mg/kg GHB in females over the estrus cycle, ovariectomized females (OVX), males and castrated (CST) males. Data is presented as mean ± SD, N = 3–5. ^*^P < 0.05 when compared to proestrus females. ^α^P < 0.05 when compared to metestrus females. ^β^P < 0.05 when compared to diestrus females

Parameter	Units	Proestrus	Estrus	Metestrus	Diestrus	OVX	Male	CST
AUC	mg*min/mL	124 ± 12.7	154 ± 20.5	126 ± 10.8	135 ± 11.5	196 ± 33.5	212 ± 65.2^* α^	189 ± 43.1
CL	mL/min/kg	8.1 ± 0.8	6.6 ± 0.9	8.0 ± 0.7	7.4 ± 0.6	5.2 ± 0.9^*α^	5.1 ± 1.4^* α β^	5.6 ± 1.3^* α^
CL_R_	mL/min/kg	3.7 ± 0.8	2.8 ± 0.4	3.9 ± 0.4	3.8 ± 0.5	2.5 ± 0.6	2.4 ± 1.2	2.8 ± 0.8
f_e_		0.45 ± 0.06	0.43 ± 0.03	0.49 ± 0.02	0.51 ± 0.08	0.47 ± 0.07	0.45 ± 0.09	0.50 ± 0.03
CL_m_	mL/min/kg	4.3 ± 0.5	3.8 ± 0.6	4.1 ± 0.3	3.6 ± 0.8	2.8 ± 0.5^*^	2.7 ± 0.5^*^	2.7 ± 0.5^*^

Renal clearance, the fraction of drug eliminated in the urine (f_e_) and metabolic clearance are presented in Tables 3 and 4. Renal clearance varied significantly between sexes and over the estrus cycle, and varied with dose. At 600 mg/kg, the highest renal clearance was observed in proestrus females, and renal clearance declined over the subsequent stages with significantly lower renal clearance in diestrus females (Table 3). Renal clearance was significantly lower in OVX and male (intact and CST) animals as compared to proestrus females. Compared to estrus stage females, renal clearance of diestrus and OVX groups were significantly lower, and consistent with renal clearance values in males. f_e_ demonstrated a similar trend to renal clearance and varied with sex and the estrus cycle, with OVX females demonstrating a significantly lower f_e_ as compared to proestrus females.

Consistent with previous data on GHB toxicokinetics, renal clearance increased with dose in males and OVX females. In cycling females renal clearance increased in metestrus and diestrus females; however, proestrus and estrus females did not demonstrate an increase in renal clearance at 1000 mg/kg. No significant differences were observed for metabolic clearance following 600 mg/kg; however, distinct differences were observed following the 1000 mg/kg dose. Cycling females had higher metabolic clearance, while males and OVX females had lower metabolic clearance, when comparing the 1000 mg/kg dose to the 600 mg/kg dose.

### MCT1 expression

**Fig. 2:**
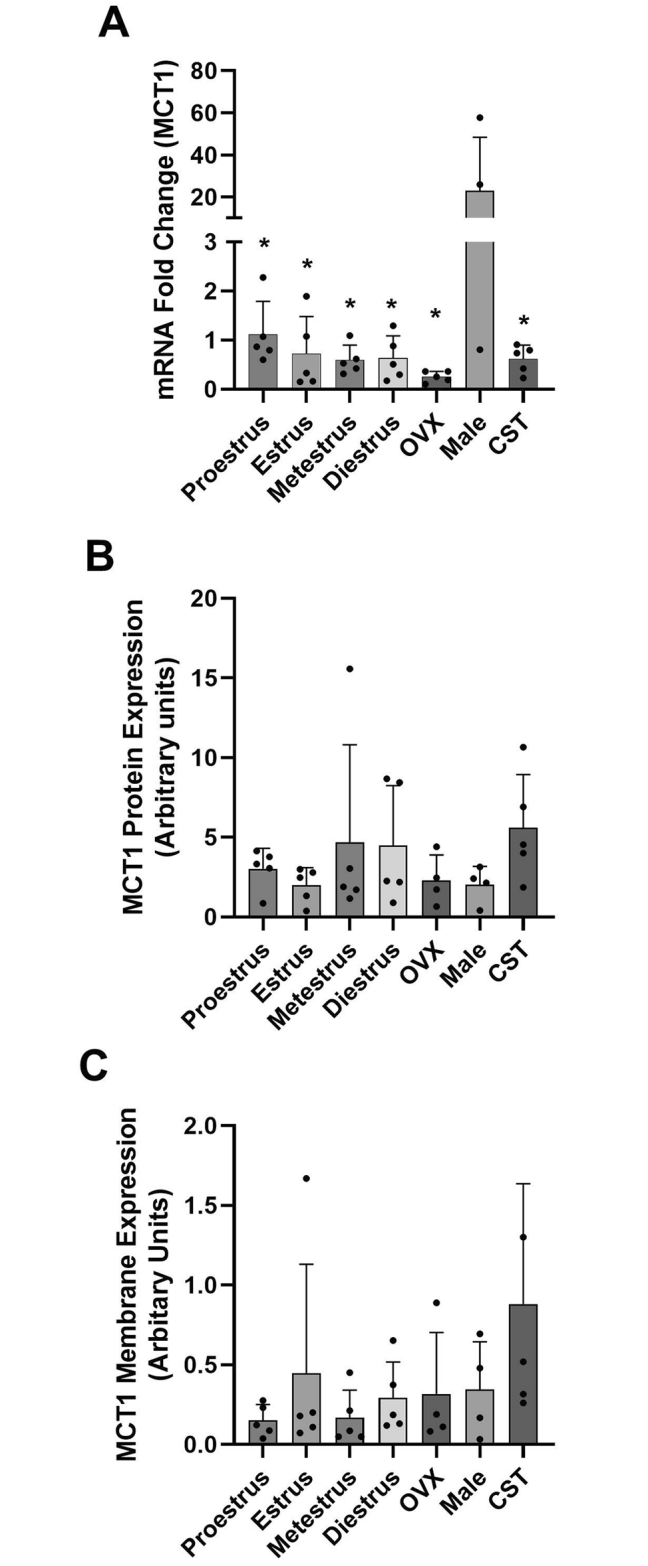
MCT1 mRNA, whole cell and membrane protein expression in the kidney of female and male rats. [**A**] Fold-change in MCT1 mRNA expression relative to proestrus females. [**B**] Whole cell MCT1 protein expression in kidney with expression normalized to GAPDH expression. [**C**] MCT1 membrane protein expression normalized to Na^+^/K^+^ ATPase expression. Data presented as mean ± SD. (n = 4–5 rats). ^*^P < 0.05 as compared to intact males

MCT1 mRNA expression (Fig. 2A) varied significantly between groups (P = 0.0049). MCT1 mRNA expression was significantly higher (20–40-fold) in male rats compared to female estrus cycle groups, while compared to OVX females, intact males had approximately 90-fold higher mRNA expression levels. CST males had significantly lower MCT1 mRNA expression as compared to intact males (Fig. 2A), with expression similar to cycling females. No significant differences were observed in mRNA expression between cycling females and OVX females. Whole cell MCT1 protein expression (Fig. 2B) varied over the estrus cycle in females with higher, but not significant, mean expression in metestrus and diestrus females. CST males had increased whole cell MCT1 expression as compared to intact males, which suggests that male sex hormones may negatively regulate overall MCT1 protein expression. Membrane MCT1 expression (Fig. 2C) varied over the estrus cycle and was lower in proestrus and metestrus females. High variability in MCT1 membrane expression was observed in estrus females due to one female with 10 times higher expression than all other estrus stage females (Fig. 2C); when this female was excluded the mean MCT1 membrane expression of estrus females was similar to that of proestrus and metestrus females. CST males had higher MCT1 mean membrane expression compared to intact males and cycling and OVX females.

### CD147 expression

**Fig. 3:**
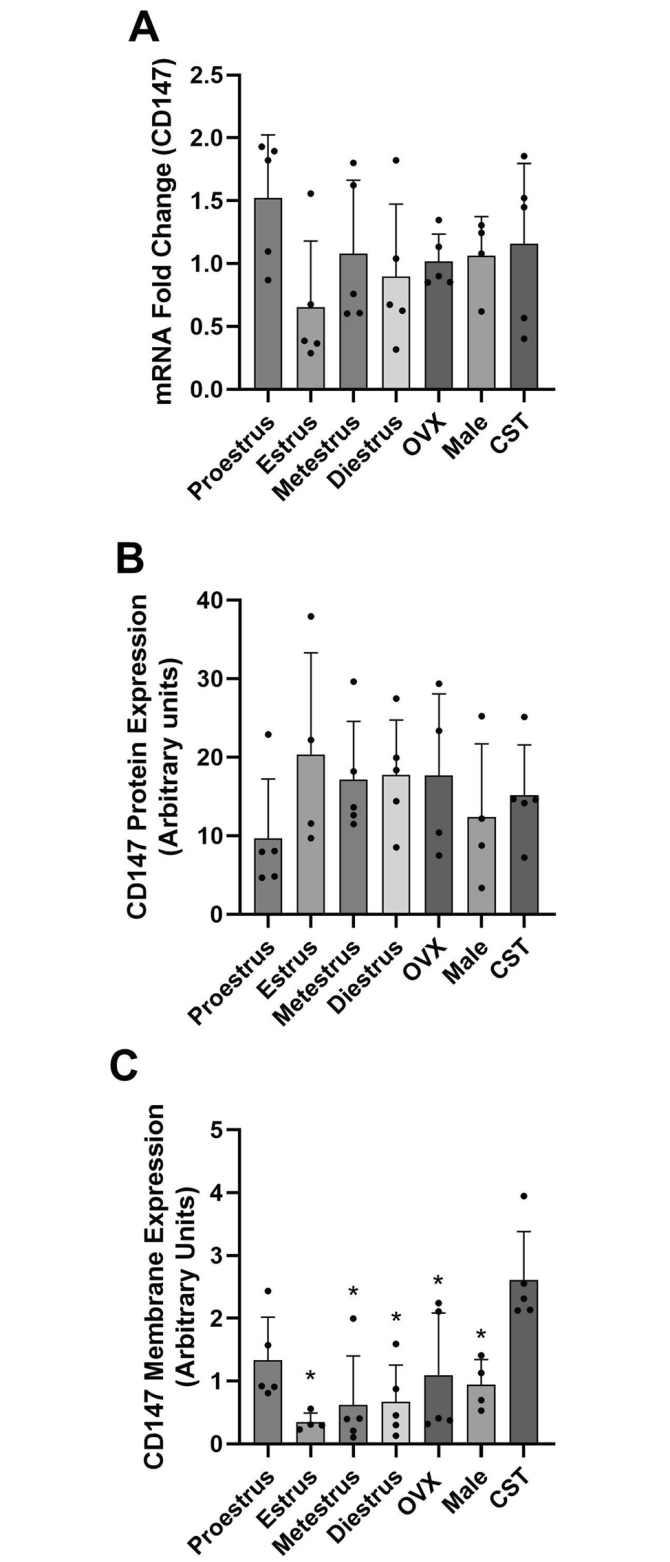
CD147 mRNA, whole cell and membrane protein expression in the kidney of female and male rats. [**A**] Fold-change in CD147 mRNA expression relative to proestrus females. [**B**] Whole cell CD147 protein expression in kidney with expression normalized to GAPDH expression. [**C**] CD147 membrane protein expression normalized to Na^+^/K^+^ ATPase expression. Data presented as mean ± SD. (n = 4–5 rats). ^*^P < 0.05 as compared to CST males

mRNA expression of the ancillary protein CD147 did not vary over the estrus cycle or in the absence of sex hormones (Fig. 3A). Whole cell CD147 expression was not significantly different between groups (Fig. 3B), but mean expression was lower in proestrus females compared to other females. Membrane CD147 protein expression demonstrated significant differences between groups (P = 0.0008). CST males had significantly higher (P < 0.05) membrane CD147 expression compared to males and OVX females, and all cycling females with the exception of proestrus stage females (Fig. 3C). Androgen removal significantly altered CD147 membrane localization. Proestrus females and OVX females trended higher than estrus, metestrus and diestrus females, and were comparable to intact males; however, these differences were not statistically significant.

### SMCT1 expression

**Fig. 4:**
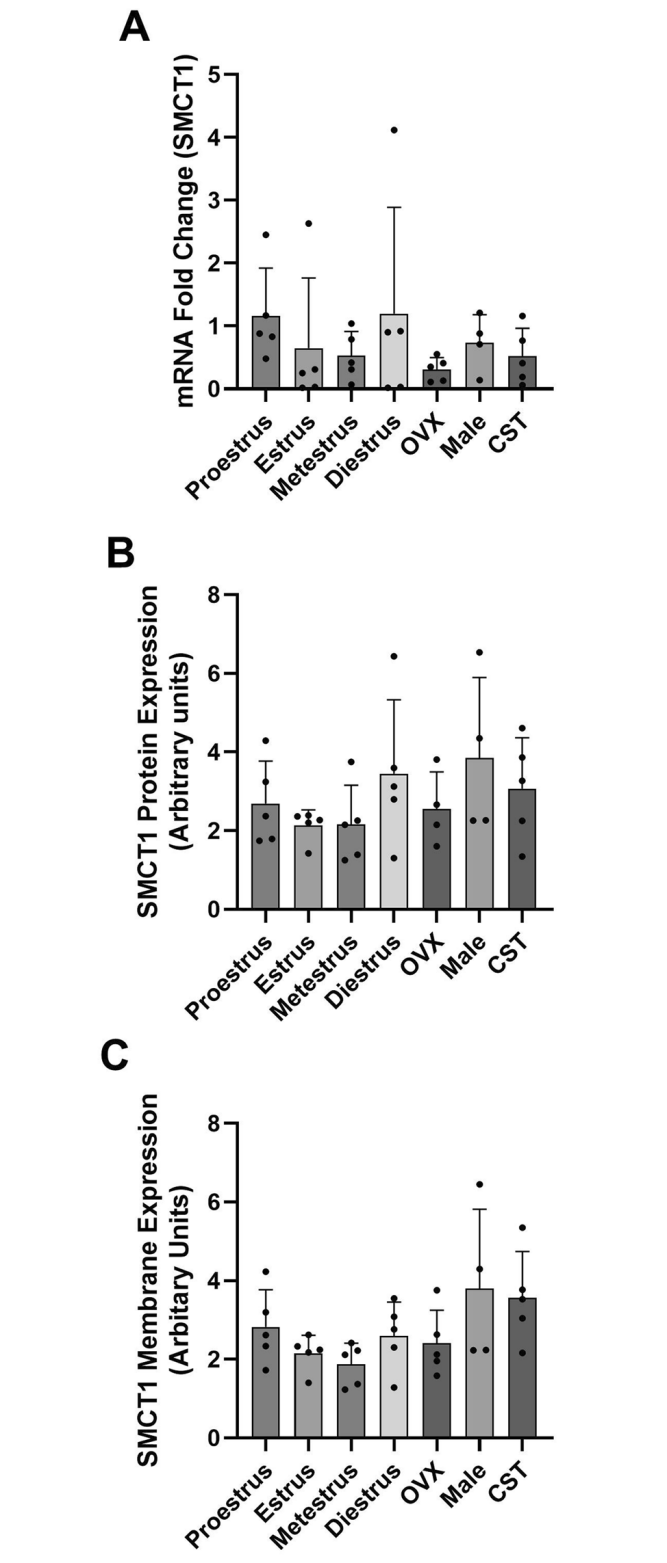
SMCT1 mRNA, whole cell and membrane protein expression in the kidney of female and male rats. [**A**] Fold-change in SMCT1 mRNA expression relative to proestrus females. [**B**] Whole cell SMCT1 protein expression in kidney with expression normalized to GAPDH expression. [**C**] SMCT1 membrane protein expression normalized to Na^+^/K^+^ ATPase expression. Data presented as mean ± SD. (n = 4–5 rats)

mRNA, whole cell and membrane expression of SMCT1 are presented in Fig. 4. No significant differences in SMCT1 mRNA or whole cell protein expression were observed between sexes, over the estrus cycle stages, or between OVX and CST rats. SMCT1 membrane expression was not statistically different between groups (P = 0.0762), which may be due to the large variability and number of animals in each group. While not statistically significant, mean SMCT1 membrane expression trended higher in intact and CST male rats suggesting that a mechanism unrelated to sex hormones may be involved.

## Discussion

Monocarboxylate transporters are involved in nutrient and drug distribution in the kidney. Alterations in renal monocarboxylate transporter expression could lead to changes in nutrient reabsorption, drug reabsorption and renal clearance. Previous studies have been demonstrated that MCTs and SMCTs are regulated in a tissue specific manner by sex hormones [22, 23, 25]; however, to our knowledge no studies have investigated the influence of the estrus cycle and sex hormones on GHB toxicokinetics and renal MCT/SMCT expression. Here, we demonstrate that GHB toxicokinetics vary over the estrus cycle in females with lower renal clearance in OVX females, as compared to proestrus females. GHB renal clearance in OVX females was similar to that in CST and intact males suggesting that female sex hormones contribute to the observed differences in toxicokinetics. Further, we demonstrated that renal MCT1 membrane expression varies over the estrus cycle, with the lowest expression observed in proestrus females, which is consistent with the observed changes in GHB renal clearance.

GHB demonstrates nonlinear toxicokinetics following iv bolus administration due to capacity-limited metabolism and renal reabsorption [7, 31]. In overdose, GHB metabolic clearance is saturated, and renal clearance becomes a significant route of elimination. The toxicokinetic profiles in the present study are consistent with previous studies reporting GHB toxicokinetics in male rats [2, 4, 32, 33]. The concentrations which lead to death in humans (post-mortem blood levels: mean 660 mg/L) are lower than those that cause death in rats; however, the concentrations in the current study are in the range of those seen in human overdose [34]. Our results demonstrate similar clearance parameters in males as compared to previous literature [33, 35]. In the present study, proestrus females demonstrated increased renal clearance and reduced plasma concentrations and exposure (AUC) at 600 mg/kg compared to other cycling females. However, at a dose of 1000 mg/kg there were no differences between cycling females suggesting saturation of GHB clearance and/or distribution pathways. Males and OVX females had lower renal and metabolic clearance at 1000 mg/kg indicating that female sex hormones reduce GHB exposure. Future studies will evaluate higher doses of GHB (up to 1500 mg/kg) and evaluate the impact of individual sex hormones on GHB toxicokinetics and GHB-induced fatality.

Sex hormone-mediated regulation of MCTs was demonstrated in Sertoli cells, skeletal muscle and brain [22, 23, 36]; however, there is minimal data in tissues involved in drug distribution. In Sertoli cells, the MCT 4 mRNA level significantly decreased with 17β-oestradiol and dihydrotestosterone treatment [23]. Administration of testosterone increased MCT1 and MCT4 protein expression in rat skeletal muscle [22]. Estrogen-related receptor alpha (ERR*α*) expression can modulate the expression of MCT1 in mice, and the induction of estrogen receptor β (ERβ) expression resulted in increased expression of MCT4 in mesothelioma cells of mice [37]. The absence of female sex hormones following ovariectomy induced a decrease in brain MCT2 expression in mice [36]. We demonstrated that MCT1 mRNA was significantly higher in male rats compared to CST males and all female groups (Fig. 2A). This indicates that androgens might play a role in the increased MCT1 mRNA expression. In contrast, whole cell and membrane MCT1 protein expression was higher in CST males compared to intact males suggesting androgens differentially regulate both transcription and translation. CD147 membrane expression demonstrated significant differences between cycling females, intact males and CST males with the highest expression observed in CST males. Proestrus females had higher CD147 membrane expression compared to other cycling females; however, expression was still less than 50% of that observed in CST males. These expression differences suggest that androgens negatively regulate CD147 membrane localization. MCT1 membrane expression had a similar pattern to membrane CD147 expression suggesting that CD147 contributes to MCT1 membrane trafficking. Taken together these results suggest that female and male sex hormones regulate monocarboxylate transporters expression and membrane trafficking; however, the influence of sex hormones is tissue-specific.

Previous studies in our laboratory have shown that MCT1 and MCT4 are regulated in a sex and sex hormone-dependent manner in the liver with lower MCT1 and MCT4 membrane expression in proestrus females, as compared to males and OVX females [25]. In the present study, we demonstrated lower MCT1 membrane expression in proestrus females compared to OVX females, and intact and CST males, which are consistent with our observed renal clearance changes. Renal clearance in proestrus females is higher than that in CST and intact males, due to lower active renal reabsorption clearance. No differences in GHB renal clearance were observed between intact and CST males despite CST males demonstrating higher renal MCT1 membrane expression. Additionally, no differences were observed in SMCT1 expression between males and females. There are two potential scenarios that may explain the observed results: [1] MCT1 membrane expression does not directly correlate with MCT1 activity; and [2] additional transporters are involved in renal reabsorption of GHB.

MCT1 activity at the plasma membrane is influenced by intra- and extra-cellular carbonic anhydrases [38]. Intracellular carbonic anhydrase II was demonstrated to increase MCT1 activity without a corresponding increase in MCT1 membrane expression. Extracellular carbonic anhydrases IV and IX were demonstrated to augment the activity of MCT1 [39–42]. Additionally, expression of carbonic anhydrase-related proteins VIII, X and XI in Xenopus oocytes were demonstrated to increase the activity of co-expressed MCT1 [43]. Sex- or sex hormone-dependent regulation of these proteins may contribute to differences in MCT1 activity in the kidney. Further studies are needed to elucidate the role of sex and sex hormones on the contribution of carbonic anhydrases to MCT1 membrane activity.

Inhibition of MCTs/SMCTs is a proposed treatment strategy for GHB overdose by inhibiting its active renal reabsorption mediated by MCTs and SMCTs [7]. Co-administration of MCT substrates and inhibitors, including lactate, increases GHB renal and total clearance in rats [17, 35, 44, 45]. Similarly, SMCT1 is involved in renal reabsorption of lactate and pyruvate, which also function as SMCT1 inhibitors [10, 16, 46]; however, to date no specific SMCT1 inhibitor has been developed [10]. The observed decreases in GHB renal clearance with lactate administration are likely due to combined inhibition of MCTs and SMCTs. After luteolin treatment, the renal and total clearances of GHB are significantly increased because of inhibition of the MCT1-mediated renal reabsorption of GHB [47]. As well, the immunosuppressive compounds AR-C117977 and AR-C122982 are potent MCT1 inhibitors [48], and co-administration with GHB lead to increased GHB renal and total clearance [49]. However, GHB renal clearance remains less than filtration clearance in the presence of MCT/SMCT inhibitors suggesting that other transporters may contribute to active renal reabsorption of GHB. Further studies are necessary to elucidate the underlying sex and sex hormone-mediated differences in MCT/SMCT transport kinetics and the potential involvement of additional transporters in GHB renal reabsorption.

In summary, we have demonstrated the GHB toxicokinetics and renal clearance vary between sexes, over the estrus cycle in females and in the absence of female sex hormones consistent with the renal expression of MCT1. Our results suggest that females may be less susceptible to GHB-induced toxicity due to decreased exposure secondary to increased renal and metabolic clearance. Future studies should evaluate the sex hormone dependent regulation of enzymes involved in GHB metabolism. Further studies are needed to comprehensively evaluate the contribution of individual sex hormones to differences in GHB toxicokinetics, monocarboxylate transporter expression, and the potential involvement of additional transporters in GHB renal reabsorption.

### Electronic supplementary material

Below is the link to the electronic supplementary material.

Supplementary Material 1

## Data Availability

The datasets used and analyzed during the current study are available from the corresponding author on reasonable request.
