# Supplementary material for: GHB toxicokinetics and renal monocarboxylate transporter expression are influenced by the estrus cycle in rats
Source: BMC Pharmacol Toxicol. 2023 Nov 2;24:58. doi: 10.1186/s40360-023-00700-y (PMC10623699; doi:10.1186/s40360-023-00700-y)

**SUPPLEMENTAL DATA**

Gel images for all western blots used to determine whole cell and membrane protein expression for MCT1, CD147 and SMCT1, as well as loading controls.

*Abbreviations:*

Proestrus – pro

Estrus – est

Metestrus – met

Diestrus – die

OVX – O

Male – M

*Whole cellular protein expression:*

MCT1

Gel1:

**
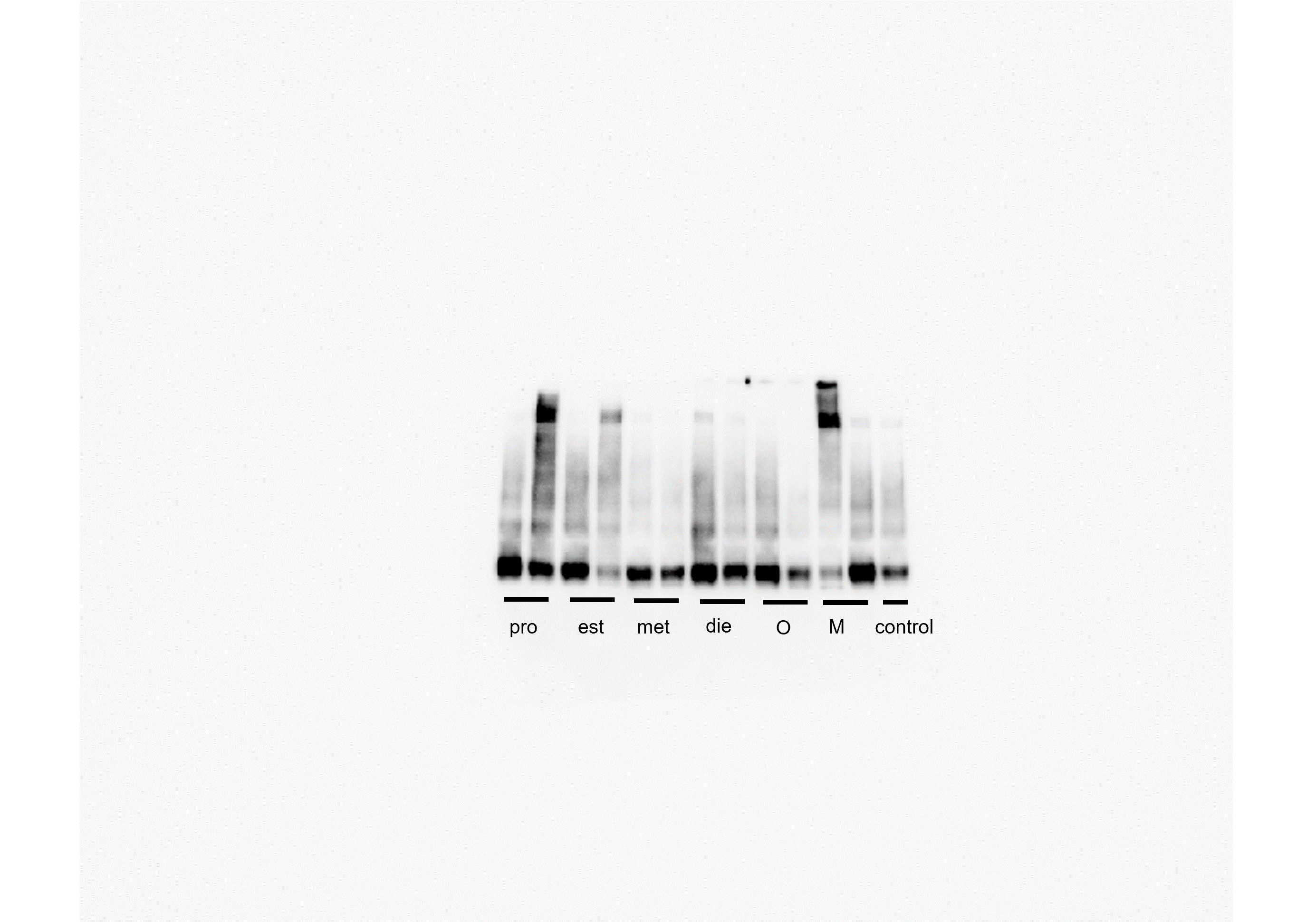
**

Gel2:


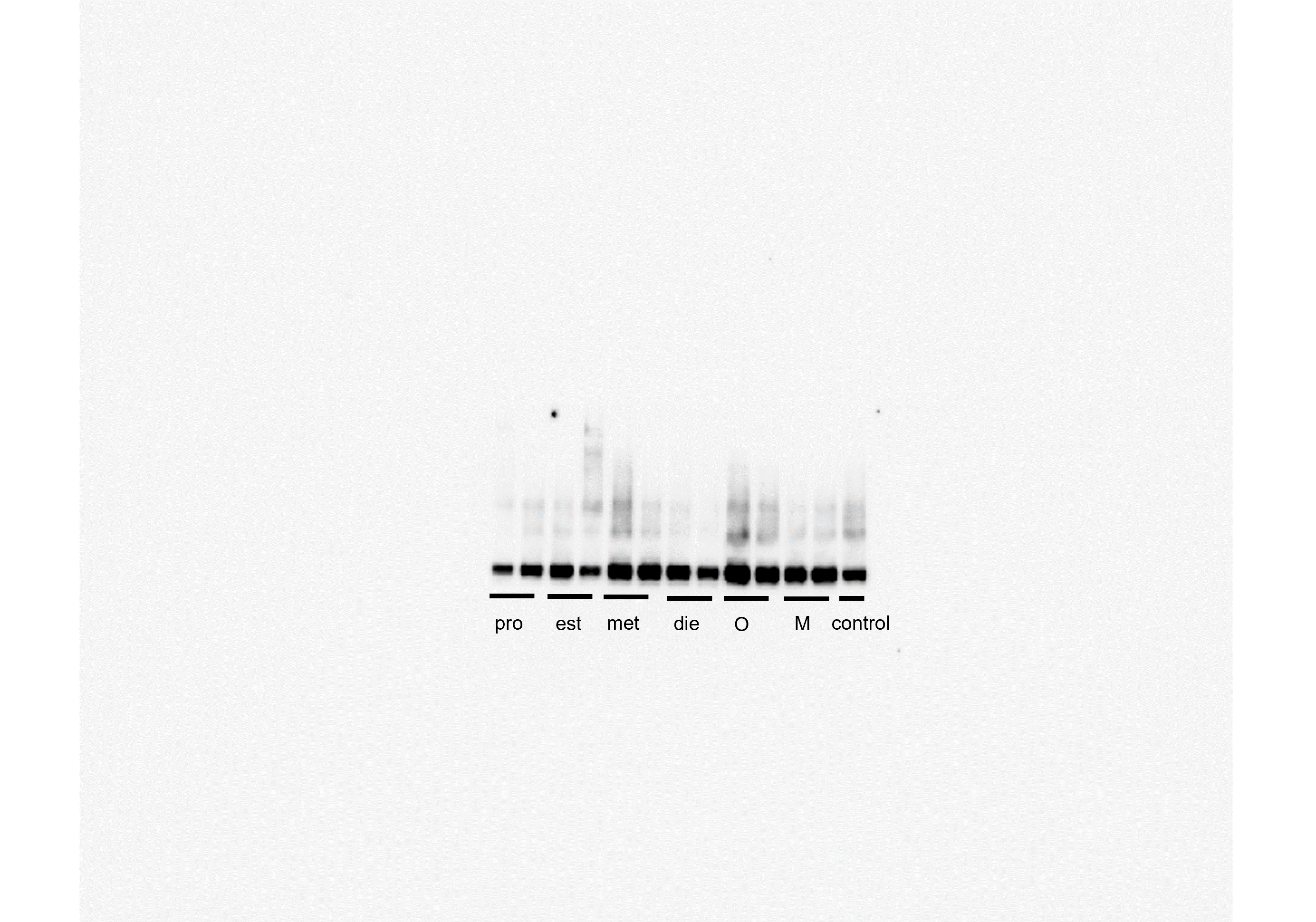


Gel3:


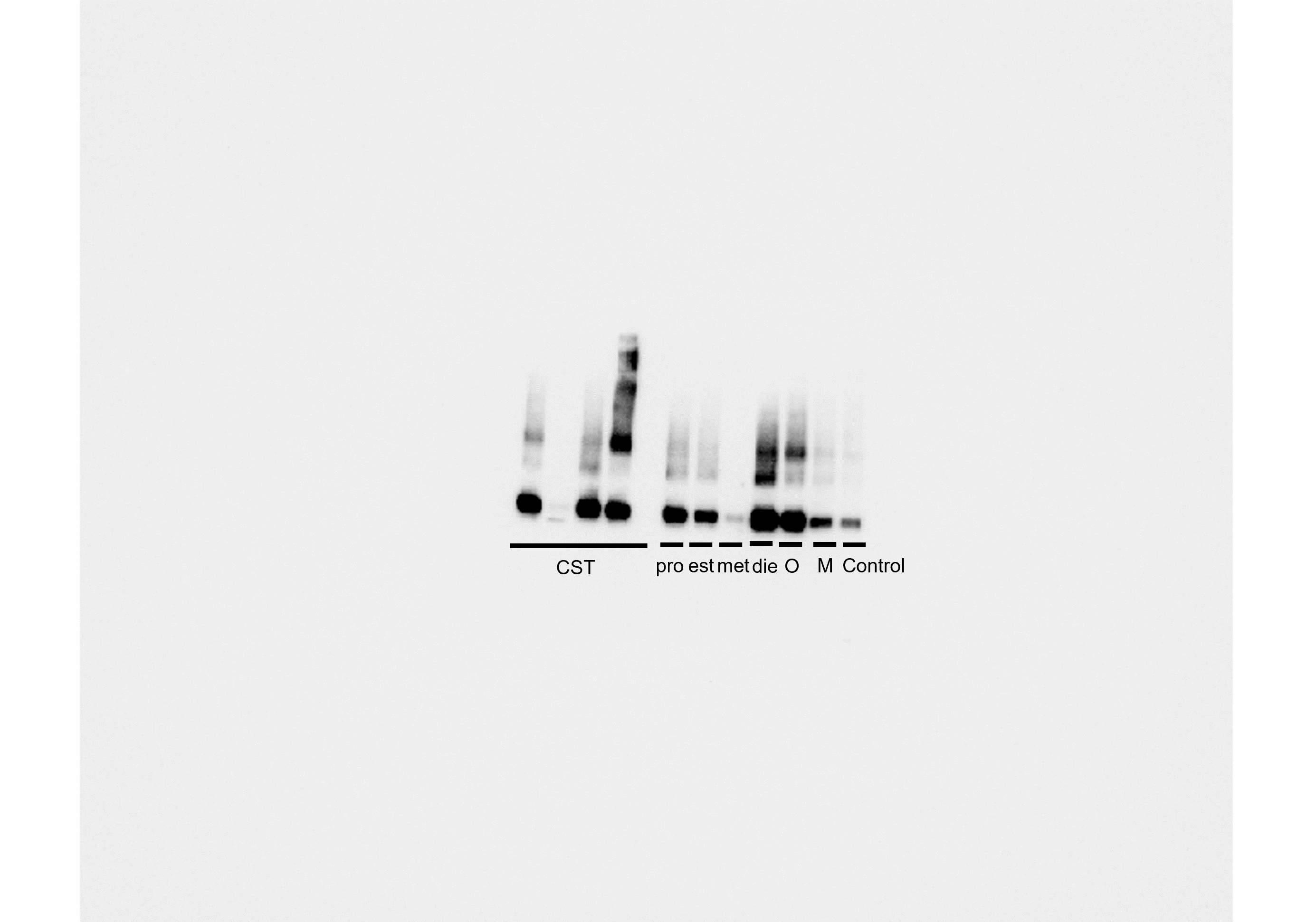


CD147:

Gel1:


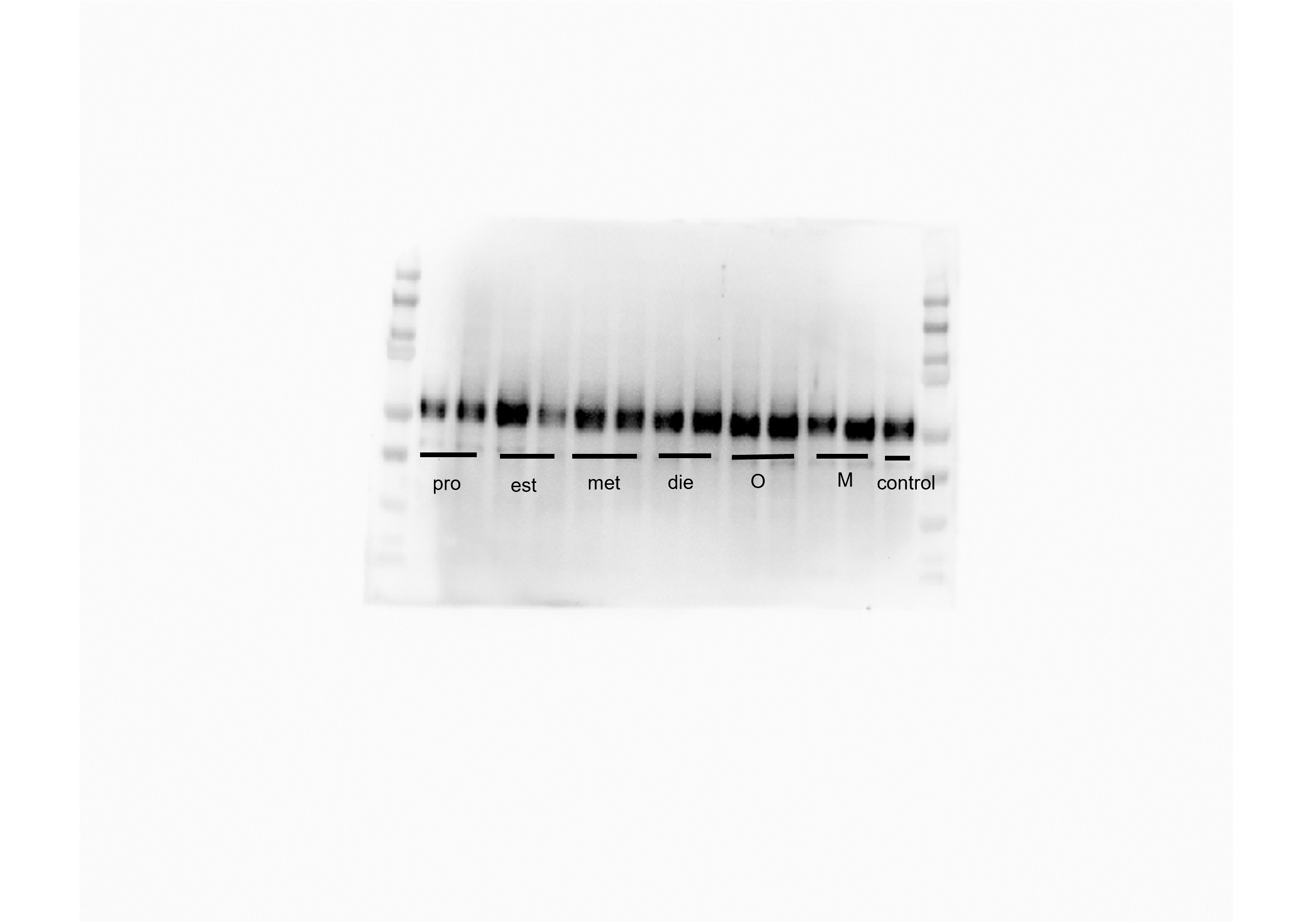


Gel2:


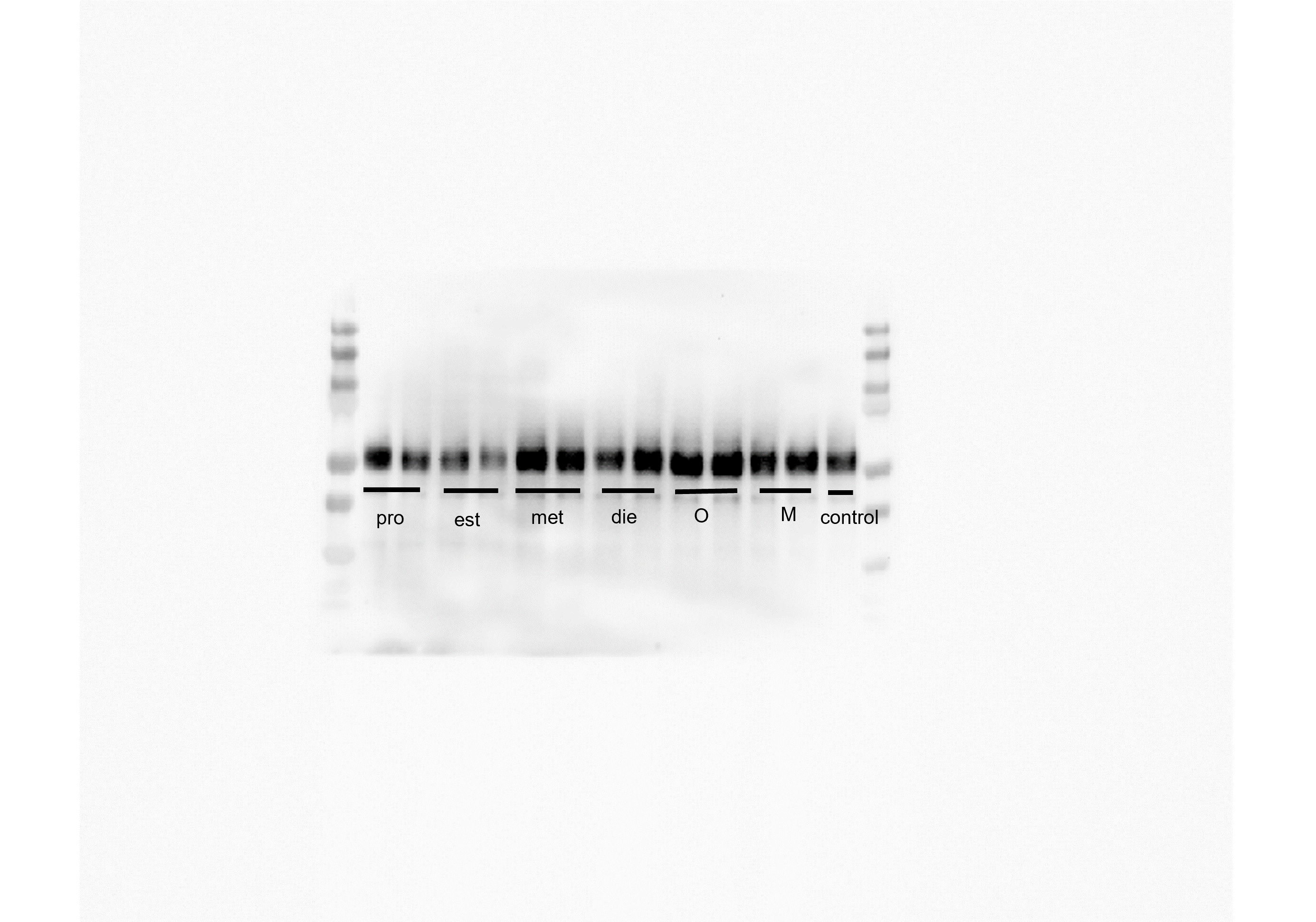


Gel3:


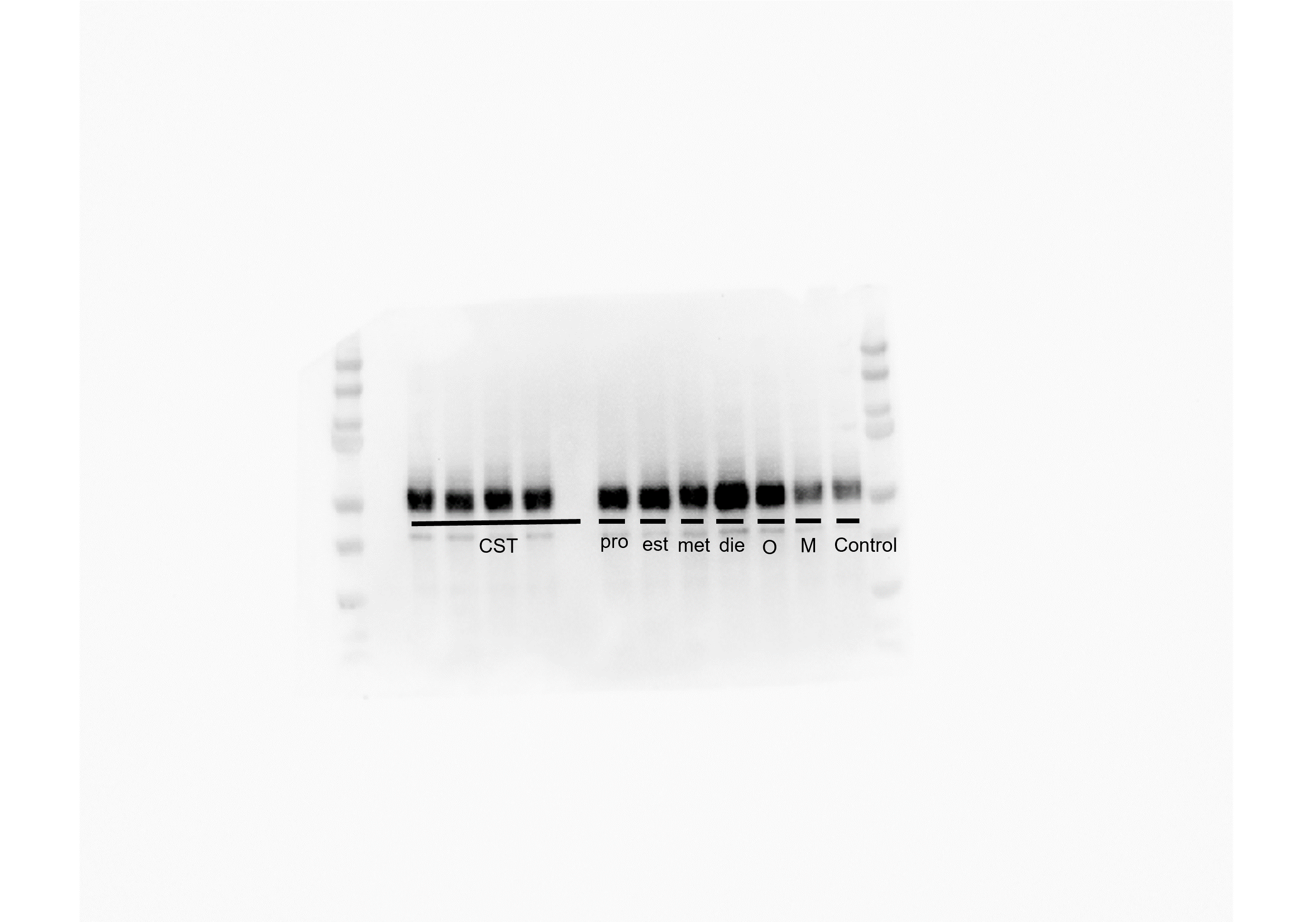


GAPDH for MCT1&CD147:

Gel1:


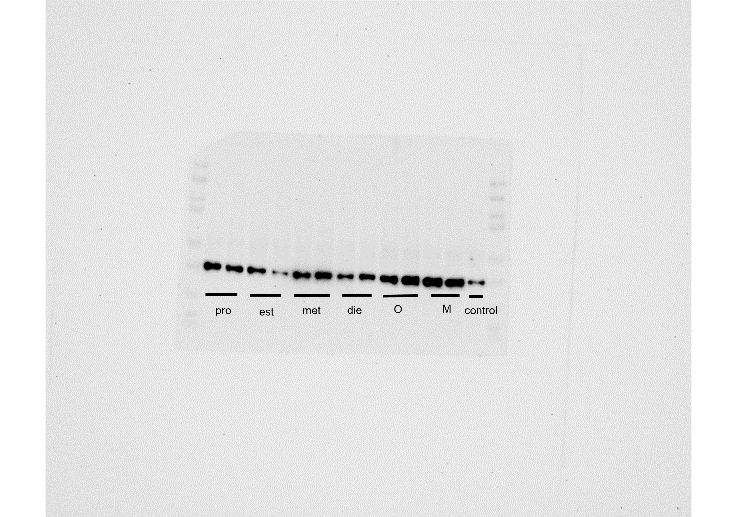


Gel2:


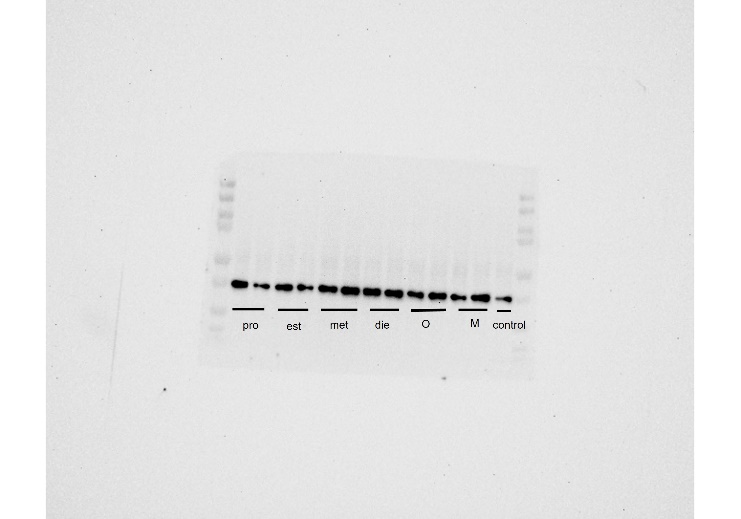


Gel3:


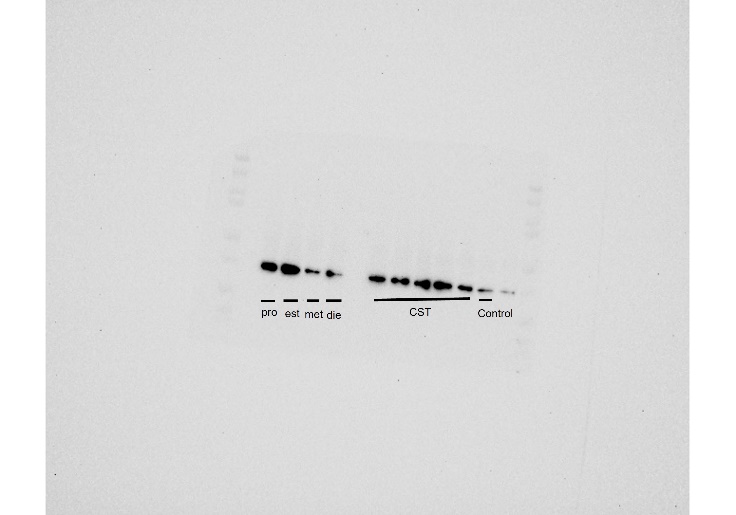


SMCT1:

Gel1:


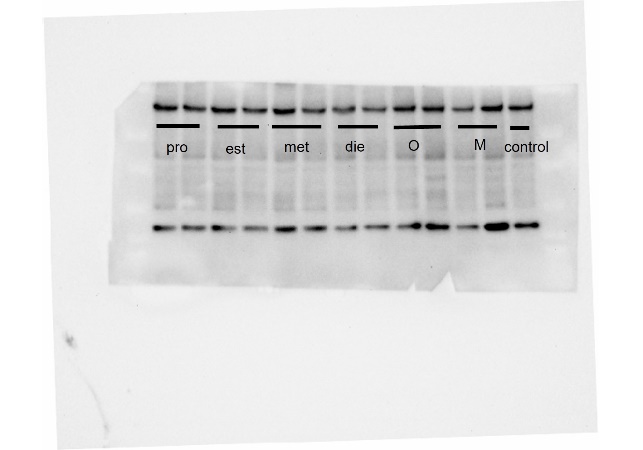


Gel2:


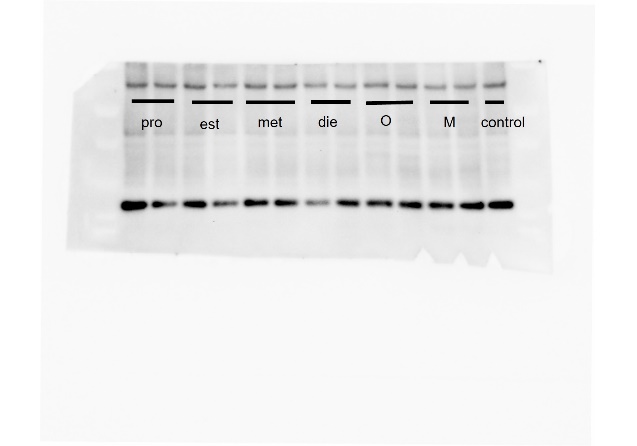


Gel3:


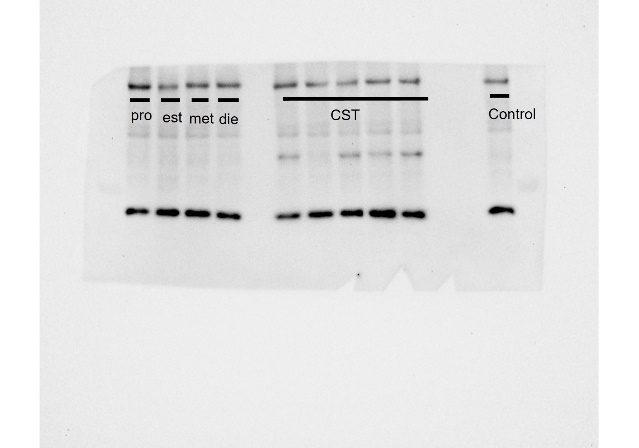


GAPDH for SMCT1:

Gel1:


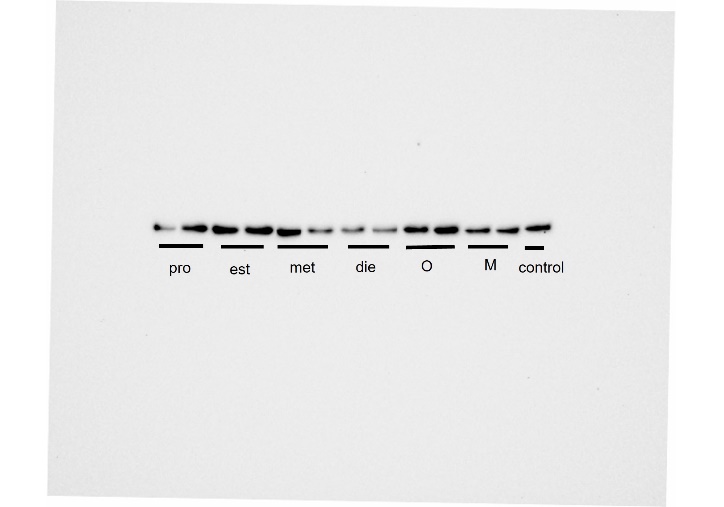


Gel2:


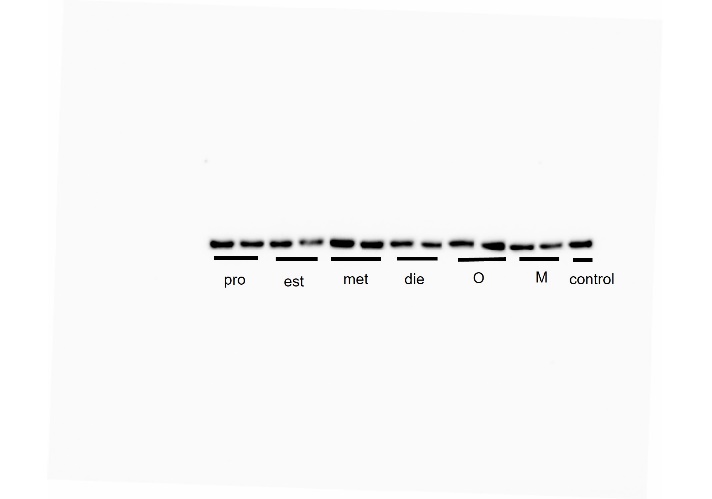


Gel3:


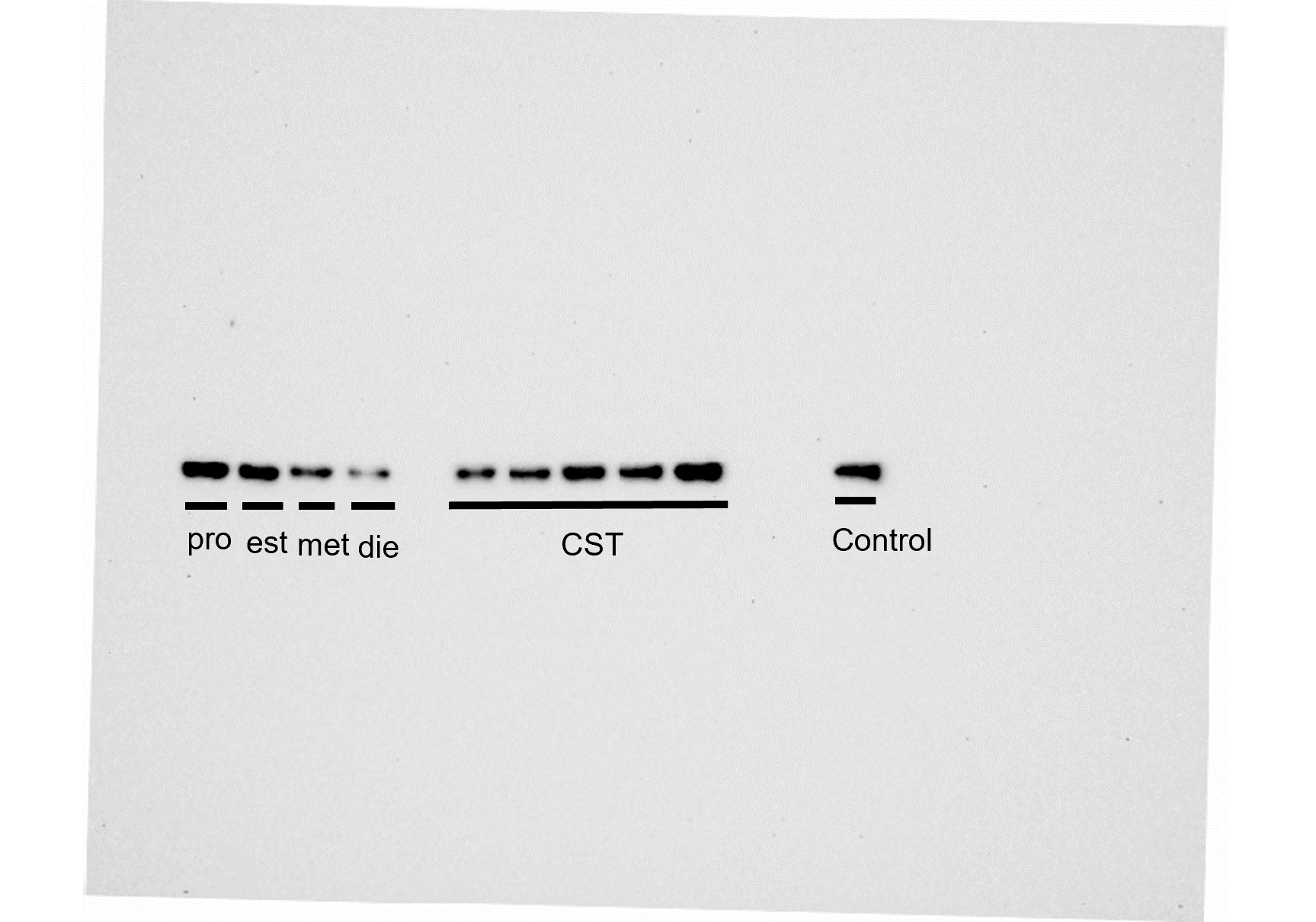


*Membrane protein expression:*

MCT1:

Gel1:


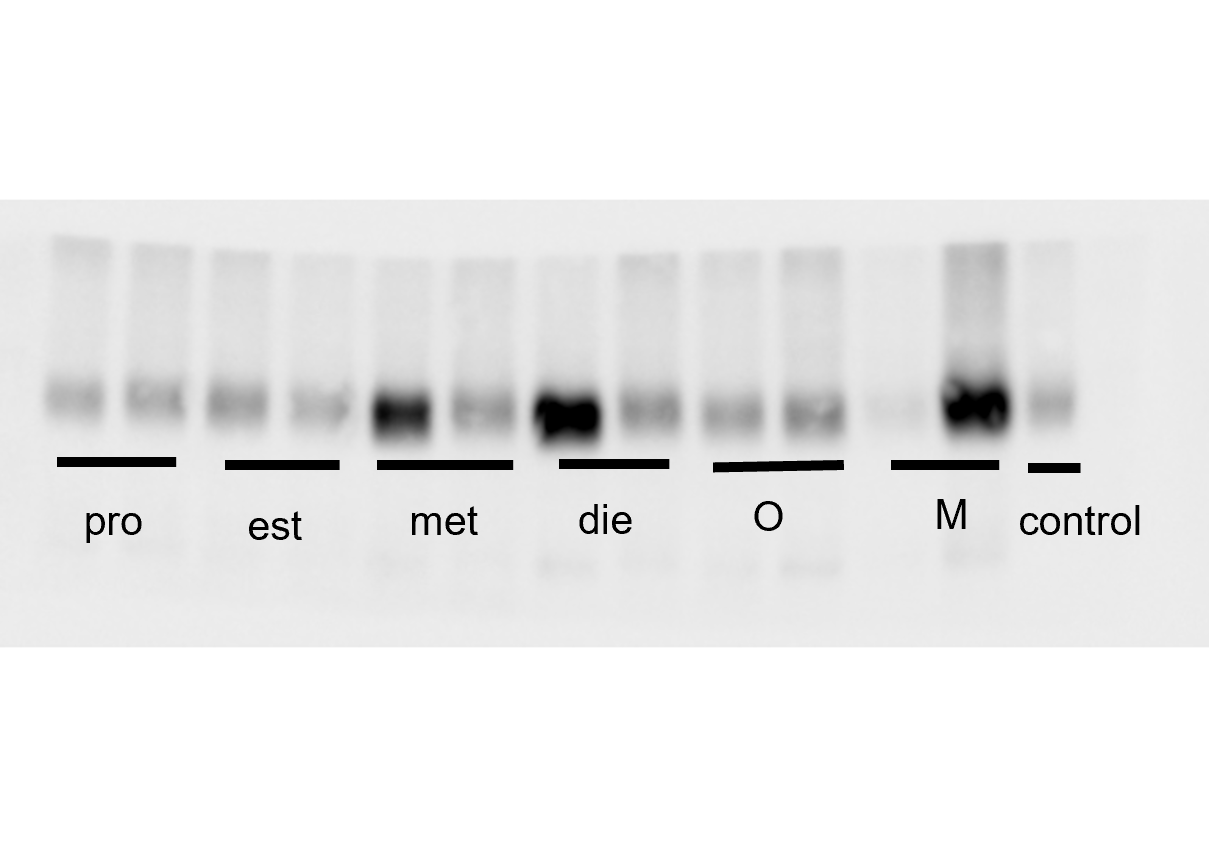


Gel2:


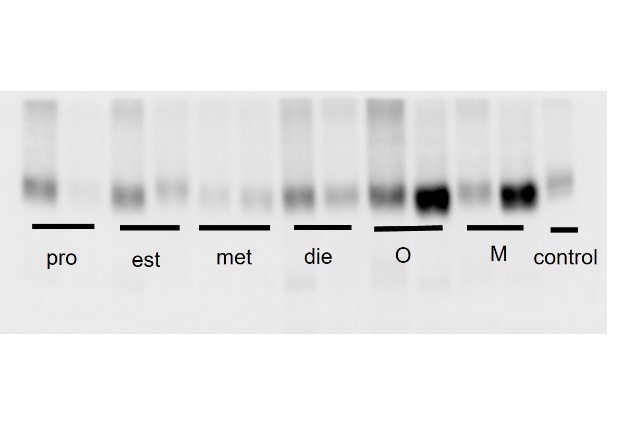


Gel3:


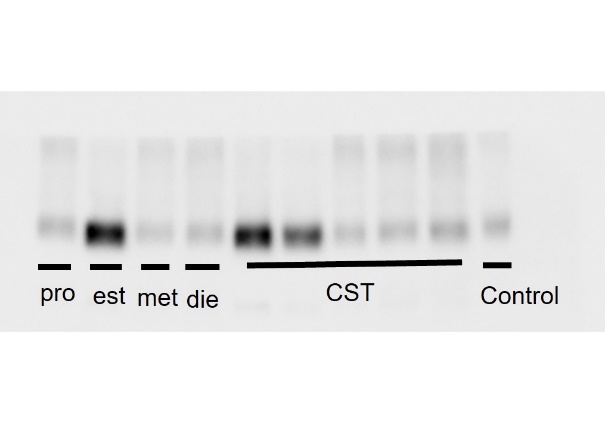


NaK for MCT1:

Gel1:


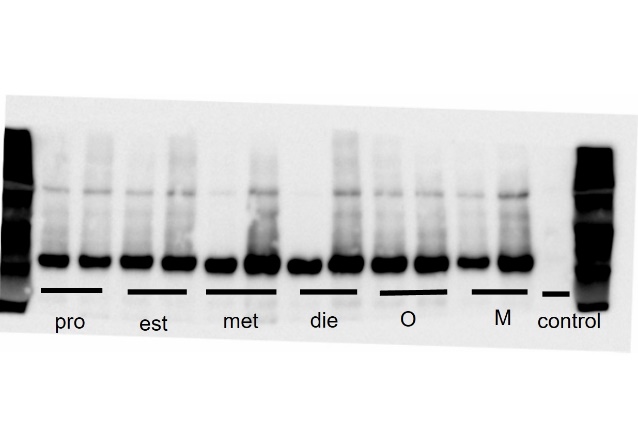


Gel2:


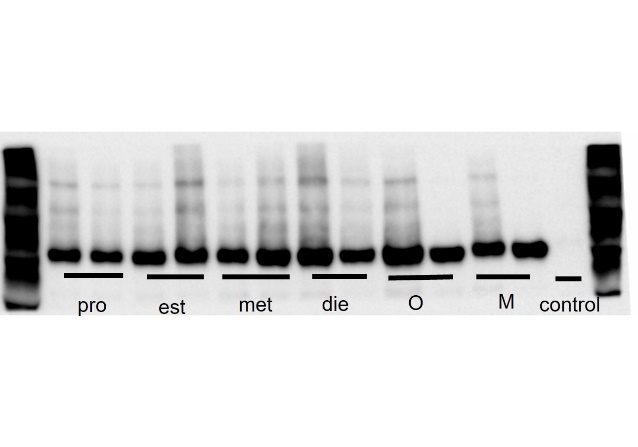


Gel3:


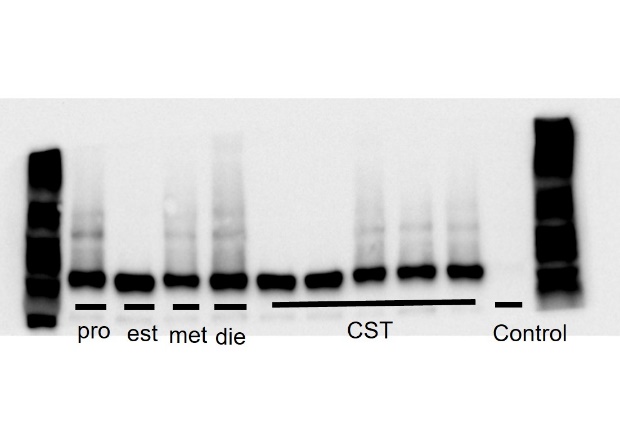


CD147:

Gel1:


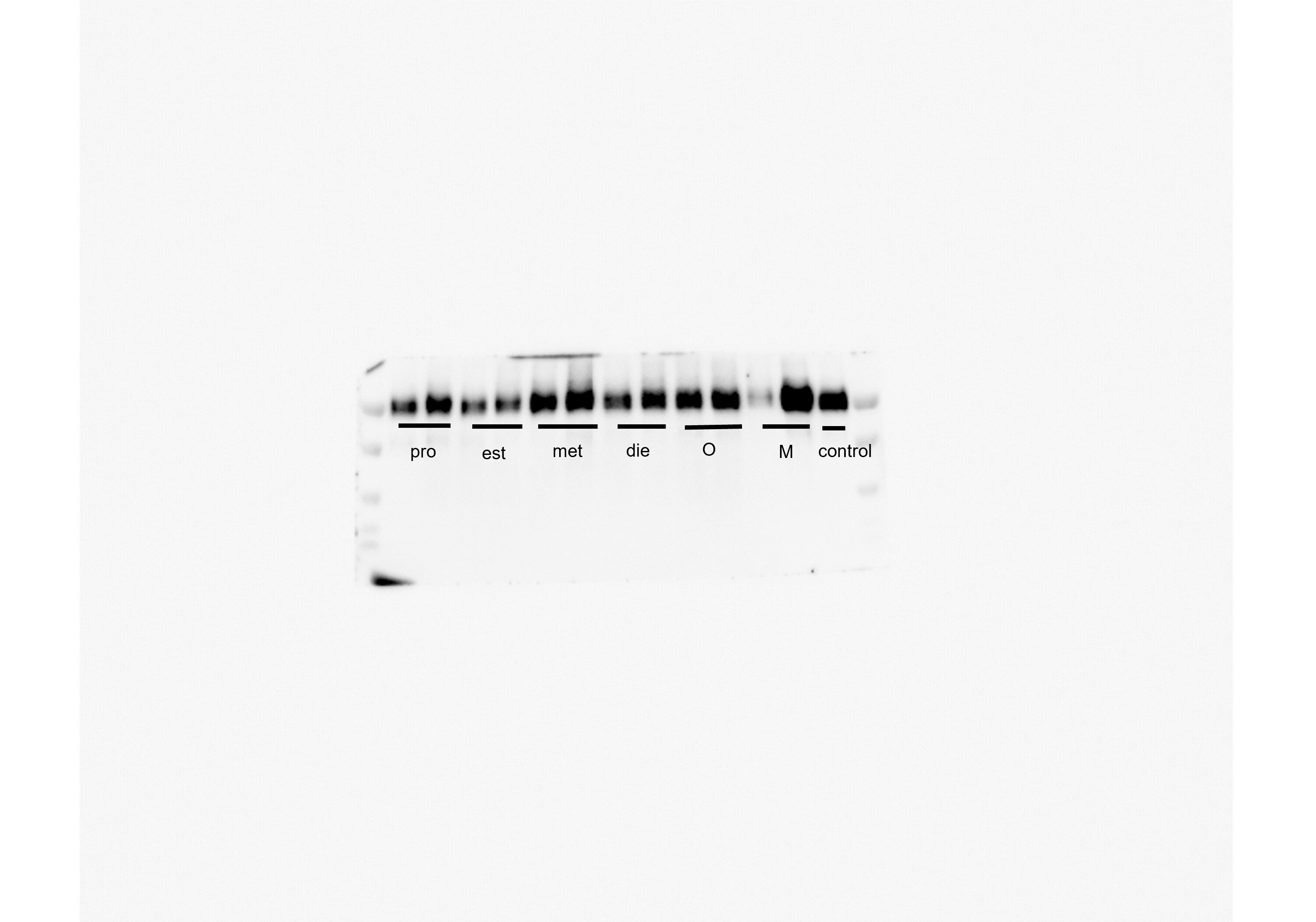


Gel2:


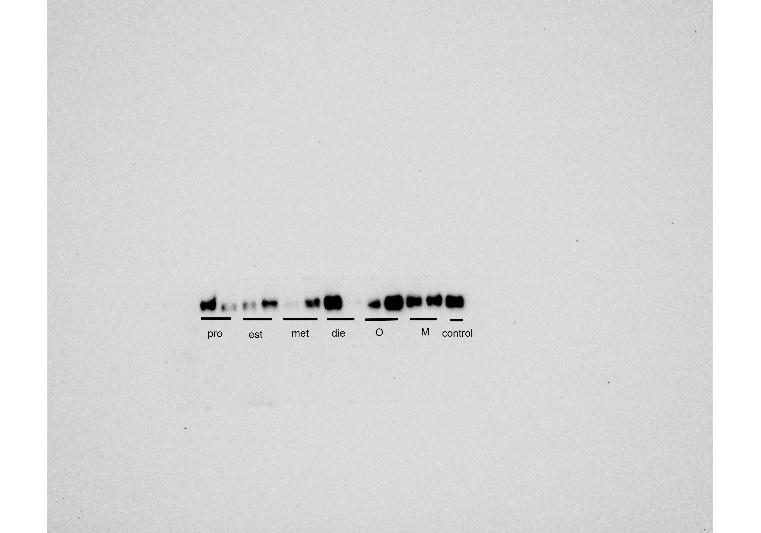


Gel3:


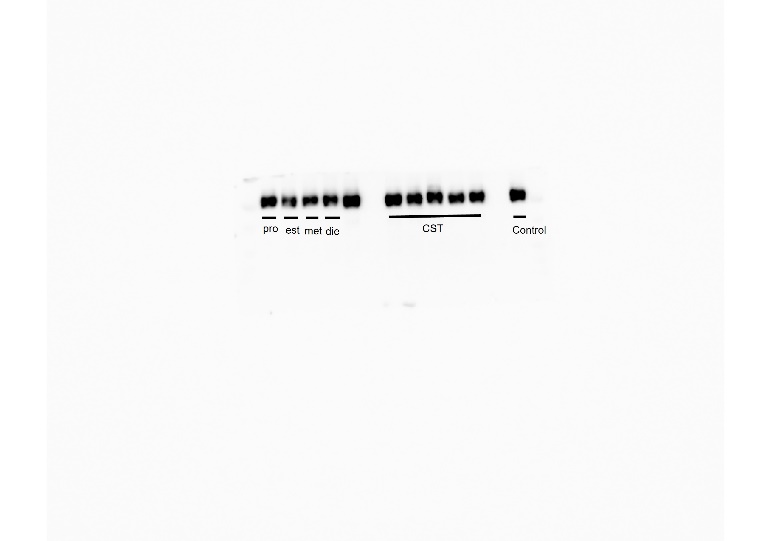


NaK for CD147:

Gel1:


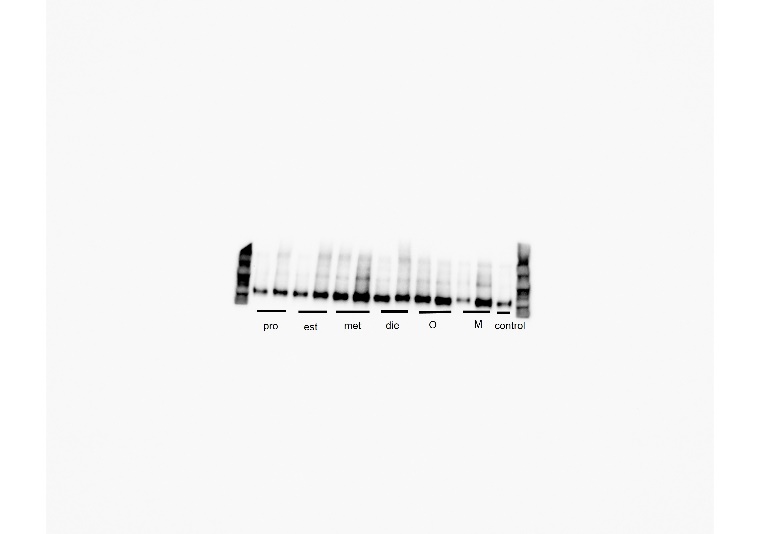


Gel2:


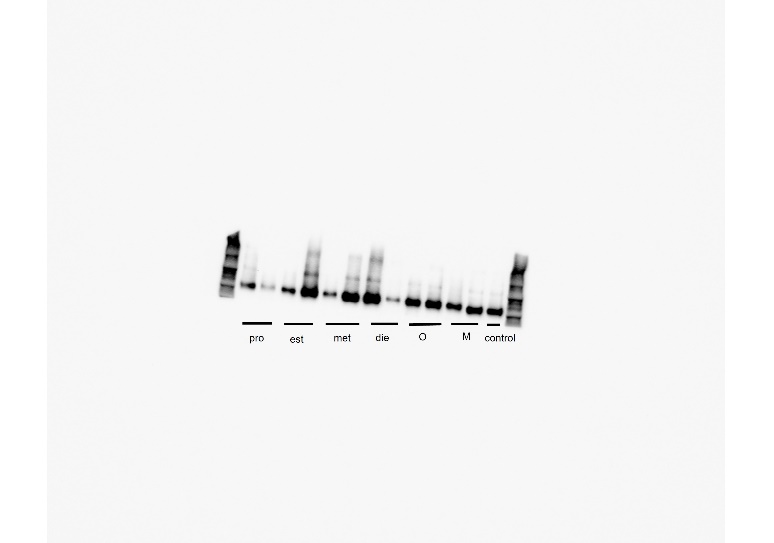


Gel3:


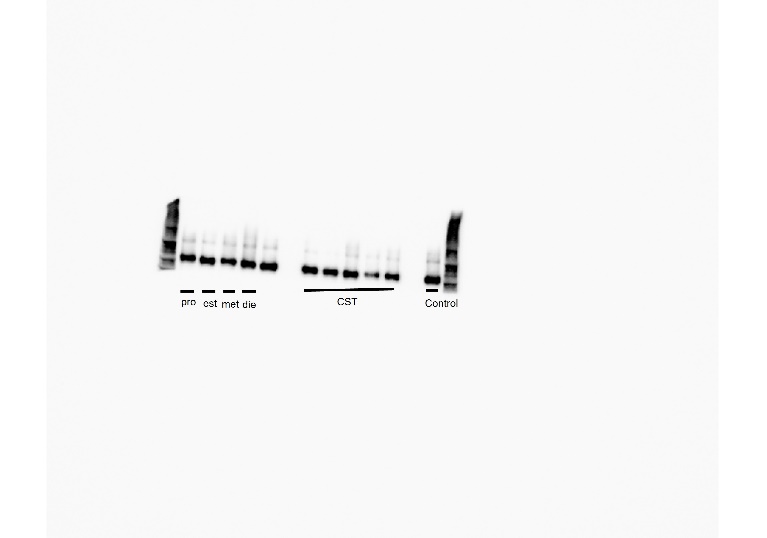


SMCT1:

Gel1:


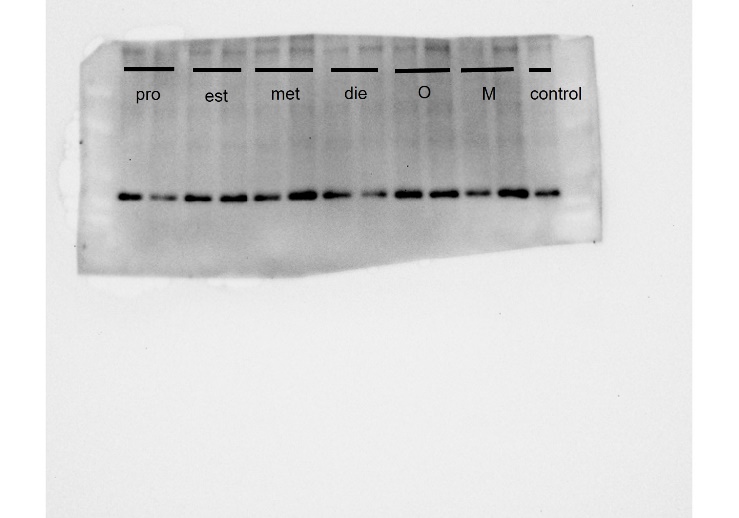


Gel2:


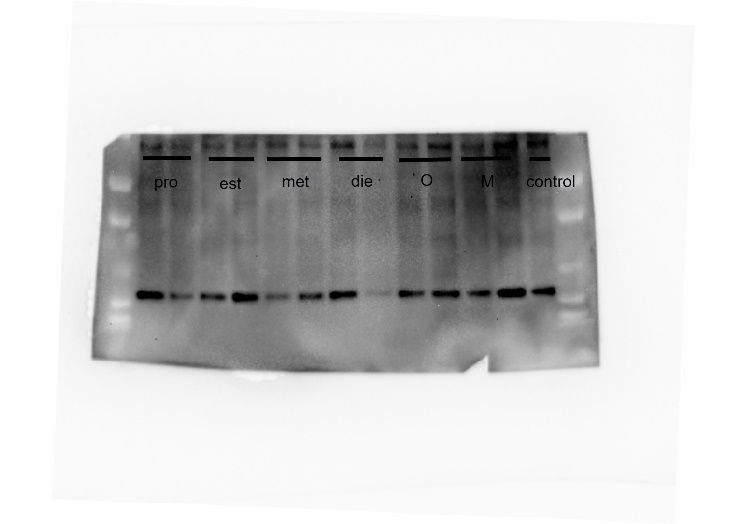


Gel3:


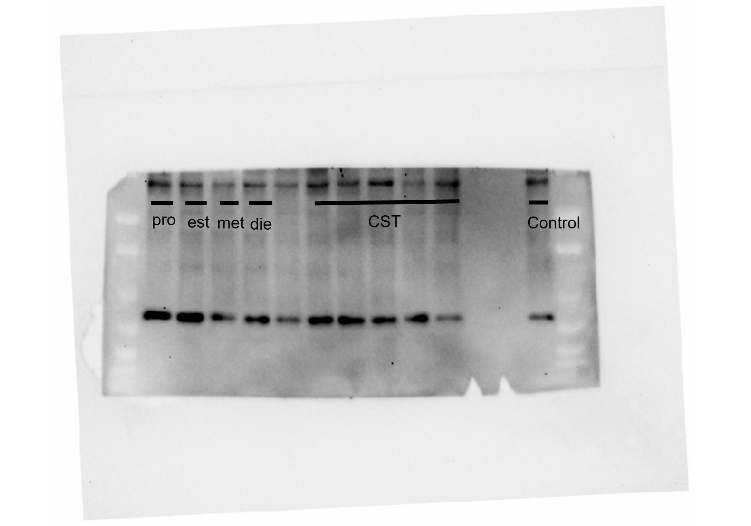


NaK for SMCT1:

Gel1:


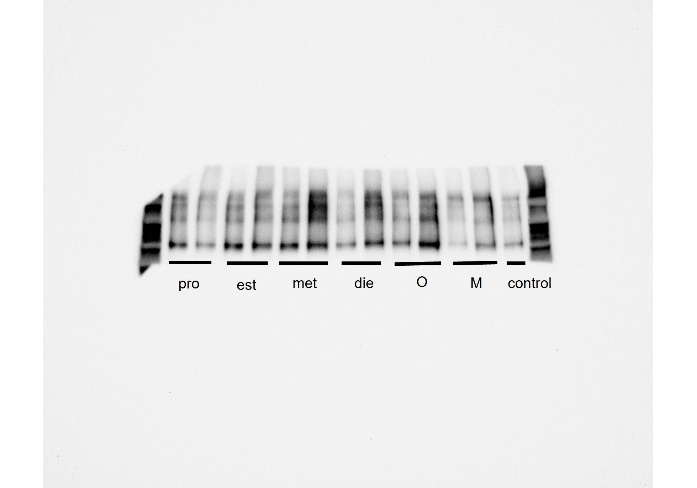


Gel2:


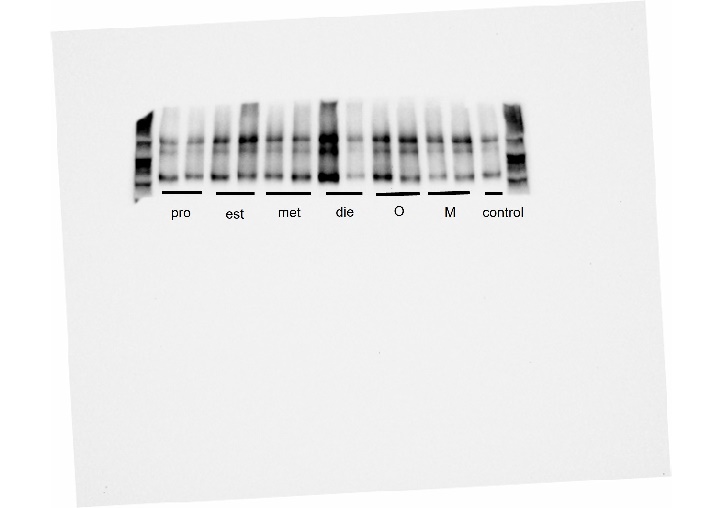


Gel3:


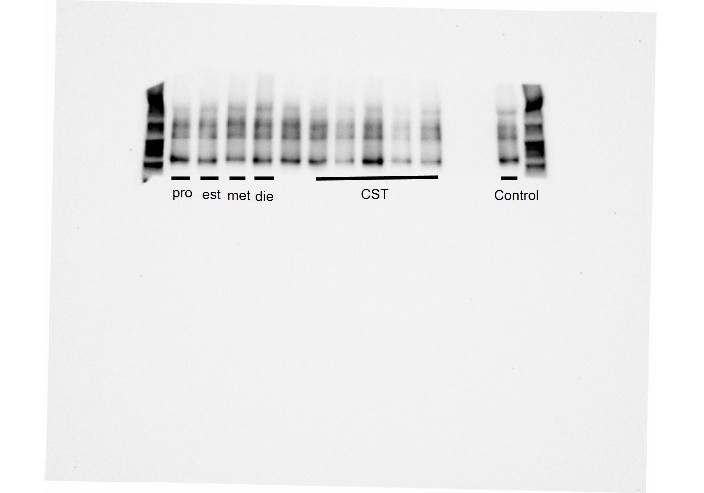

Supplement: Supplementary file 1 — Supplementary Material 1 [file 40360_2023_700_MOESM1_ESM.docx]
